# Clinical Risk Models, Inflammatory–Nutritional Indices and a Neural-Network Survival Model in Metastatic Renal Cell Carcinoma: A Single-Center Retrospective Study

**DOI:** 10.3390/jcm15218262

**Published:** 2026-10-23

**Authors:** Mürsel Sali, Enes Yigit, Sevgi Dilan Ardıç, Seda Sali, Alper Coskun, Ender Eren Ozcelik, Buket Erkan Özmarasalı, Ali Aktaş, Ahmet Bilgehan Sahin, Erdem Çubukçu, Türkkan Evrensel, Adem Deligönül

**Affiliations:** 1 Department of Medical Oncology, School of Medicine, Bursa Uludağ University, 16059 Bursa, Türkiye; endereren@uludag.edu.tr (E.E.O.); buketerkan91@gmail.com (B.E.Ö.); aliaktas@uludag.edu.tr (A.A.); absahin@uludag.edu.tr (A.B.S.); erdemcubukcu@uludag.edu.tr (E.Ç.); evrensel@uludag.edu.tr (T.E.); ademd@uludag.edu.tr (A.D.); 2 Department of Electrical and Electronics Engineering, Faculty of Engineering, Bursa Uludağ University, 16059 Bursa, Türkiye; enesyigit@uludag.edu.tr (E.Y.); dilanardic8@gmail.com (S.D.A.); 3 Department of Medical Oncology, Bursa City Hospital, 16250 Bursa, Türkiye; seda_ist1987@hotmail.com; 4 Department of Medical Oncology, Bursa Yuksek Ihtisas Training and Research Hospital, University of Health Sciences, 16310 Bursa, Türkiye; alpercoskun@uludag.edu.tr

**Keywords:** metastatic renal cell carcinoma, IMDC, MSKCC, pan-immune-inflammation value, HALP score, nephrectomy, neural network, machine learning, prognostic model

## Abstract

**Background/Objectives:** In metastatic renal cell carcinoma (mRCC), the International Metastatic Renal Cell Carcinoma Database Consortium (IMDC) and Memorial Sloan Kettering Cancer Center (MSKCC) risk models remain the primary tools for prognostic stratification but may not fully capture outcome heterogeneity in the contemporary treatment era. Systemic inflammatory–nutritional biomarkers such as the pan-immune-inflammation value (PIV) and the hemoglobin–albumin–lymphocyte–platelet (HALP) score have emerged as complementary, readily available prognostic indicators, yet their added value alongside established risk models, nephrectomy status, and modern predictive approaches such as machine learning remains poorly defined in real-world cohorts. This study jointly evaluated established risk models, inflammatory–nutritional biomarkers, and an exploratory neural-network survival model for prognostication in mRCC. **Methods:** In this retrospective, single-center cohort study, 96 patients with mRCC diagnosed and treated between January 2010 and January 2025 were evaluated, with clinical outcomes retrospectively reviewed up to a data cut-off of 17 November 2025. Clinicopathological characteristics, IMDC and MSKCC risk classification, nephrectomy status, and baseline PIV and HALP values were assessed for their association with progression-free survival (PFS) and overall survival (OS) using Kaplan–Meier and multivariable Cox regression analyses. As an exploratory analysis, a dual-head neural network predicting both the risk of death and survival time was developed; feature screening and hyperparameter tuning were nested within stratified 5-fold cross-validation repeated 10 times, and the network was benchmarked on identical splits against IMDC, MSKCC, Cox, elastic-net Cox and random survival forest models. **Results:** At a median follow-up of 82.6 months, 77 PFS events and 57 deaths had occurred; median PFS and OS were 17.8 and 46.3 months, respectively. IMDC risk classification was significantly associated with both PFS and OS, whereas MSKCC risk classification was associated with OS but not PFS; PIV and HALP were associated with OS only. In multivariable analyses, nephrectomy status remained independently associated with both PFS and OS, whereas IMDC and MSKCC classifications remained associated with OS in separate adjusted models, although the MSKCC estimates should be interpreted cautiously because of the relatively small poor-risk subgroup. The neural network achieved a nested cross-validated C-index of 0.685 (95% CI, 0.608–0.764), comparable to a Cox model with IMDC risk group and nephrectomy status (0.692) and a random survival forest (0.695), numerically higher than the IMDC risk group alone (0.629) and higher than the MSKCC risk group alone (0.594). Its integrated Brier score was 0.189, compared with 0.164 for the IMDC-plus-nephrectomy Cox model, 0.183 for the random survival forest, and 0.221 for the Kaplan–Meier reference; its calibration slope was 0.54, indicating overly extreme predictions. **Conclusions:** Established risk models remained informative, particularly for OS, while nephrectomy status was the most consistent prognostic factor across conventional and neural-network analyses; PIV and HALP were associated with overall survival in unadjusted analyses but did not show independent prognostic value. An exploratory neural-network model showed discrimination comparable to conventional Cox-based models; however, its individualized survival-probability estimates had greater prediction error than those of the IMDC-plus-nephrectomy Cox model and were poorly calibrated in slope. Recalibration and external validation in larger, contemporary cohorts are required before clinical implementation.

## 1. Introduction

Metastatic renal cell carcinoma (mRCC) is a clinically and biologically heterogeneous disease characterized by substantial variability in treatment response and survival outcomes [1]. The therapeutic landscape of mRCC has markedly evolved with the incorporation of immune checkpoint inhibitor (ICI)-based combinations, including dual ICI and ICI–vascular endothelial growth factor receptor tyrosine kinase inhibitor (VEGFR-TKI) strategies, which currently constitute the backbone of first-line systemic treatment [1,2]. Long-term follow-up of pivotal randomized trials has confirmed durable progression-free survival (PFS) and overall survival (OS) benefits with these approaches; nevertheless, a considerable proportion of patients still experience early progression or limited treatment benefit [2,3].

The International Metastatic Renal Cell Carcinoma Database Consortium (IMDC) model, originally developed in patients with mRCC receiving vascular endothelial growth factor–targeted therapy and subsequently externally validated in a population-based cohort, remains the most widely used contemporary clinical–laboratory prognostic classification in mRCC [4,5] and continues to guide risk stratification in the contemporary immunotherapy era [6,7]. Recent real-world data have confirmed that IMDC risk categories retain prognostic value in patients receiving first-line immune-based combinations, although their discriminative performance may vary according to treatment strategy and outcome measure [7]. Such findings indicate that substantial prognostic heterogeneity persists within conventional risk categories and support the investigation of complementary biomarkers capable of refining individual risk assessment [6,7]. The Memorial Sloan Kettering Cancer Center (MSKCC) model, originally developed by Motzer et al., represents another established prognostic framework that stratifies patients with metastatic RCC into favorable-, intermediate-, and poor-risk groups according to pretreatment clinical and laboratory characteristics [8]. Both IMDC and MSKCC remain useful for prognostic stratification in modern mRCC practice; however, their discriminatory capacity may be attenuated over time, particularly in patients receiving immune checkpoint inhibitor-based therapy [9].

Systemic inflammation, immune competence, and nutritional status are increasingly recognized as important host-related determinants of cancer progression and treatment outcomes. In this context, routinely available peripheral blood parameters have gained interest as inexpensive and reproducible prognostic biomarkers that may complement established clinical–laboratory prognostic models [10]. Composite indices that integrate hematological, inflammatory, and nutritional variables may be particularly informative because they capture several dimensions of the tumor–host interaction within a single measure [10,11].

The hemoglobin–albumin–lymphocyte–platelet (HALP) score integrates anemia, nutritional status, and immune-inflammatory parameters, and recent data have demonstrated an association between lower HALP values and inferior PFS and OS in patients with mRCC treated with nivolumab [10]. Similarly, the pan-immune-inflammation value (PIV), which incorporates neutrophil, platelet, monocyte, and lymphocyte counts, has shown prognostic relevance in mRCC patients receiving immune checkpoint inhibitors [11]. Among the available blood-based indices, PIV and HALP were selected because they capture complementary inflammatory, immune, hematologic, and nutritional domains using routinely available pretreatment laboratory measurements. PIV integrates neutrophil, monocyte, platelet, and lymphocyte counts and was originally described by Fucà et al. [12]; its prognostic relevance across different malignancies was subsequently evaluated in a pan-cancer meta-analysis [13]. HALP, originally described by Chen et al. [14], combines hemoglobin, albumin, lymphocyte, and platelet values. Although other established indices, including NLR, SII, and PNI, also have prognostic data in mRCC, they were not additionally evaluated to limit multiple testing and model complexity in this modest-sized cohort; therefore, the selection of PIV and HALP should not be interpreted as evidence of their superiority. Previous mRCC evidence for PIV included patients treated with nivolumab in the second line and beyond, whereas the present study assessed pretreatment indices at first-line treatment initiation [11]. Despite increasing interest in these biomarkers, direct evaluation of multiple inflammatory and nutritional indices within the same real-world mRCC cohort remains limited, particularly when considered together with established prognostic factors such as IMDC and MSKCC risk classification [6,7,8,9,10,11,15].

Although established prognostic systems such as IMDC and MSKCC remain useful for risk stratification in mRCC, conventional models may not fully capture complex and nonlinear interactions among individual patient characteristics [16,17]. Neural-network-based survival models offer an alternative approach by integrating multiple clinical variables to generate individualized risk and survival estimates [16]. In mRCC, machine learning models incorporating routinely available clinicopathological variables have demonstrated promising performance for overall survival prediction and have shown greater discriminatory performance than conventional prognostic systems in selected cohorts [17]. Nevertheless, the generalizability of AI-based prognostic models remains an important concern, emphasizing the need for rigorous validation before their implementation in routine clinical practice [18]. The primary objective of this study was to evaluate the associations of established clinical–laboratory risk models and the PIV and HALP indices with PFS and OS using conventional survival analyses. As an exploratory secondary objective, we evaluated a neural-network survival model integrating established risk classifications with routinely available clinical, laboratory, and inflammatory–nutritional variables to generate individualized risk and survival estimates, and assessed whether it provided incremental prognostic value over established risk models and conventional Cox regression when all models were evaluated on identical cross-validation splits.

## 2. Materials and Methods

### 2.1. Study Design and Patient Population

This retrospective, single-center cohort study included patients with mRCC who were diagnosed and initiated first-line systemic therapy at the Department of Medical Oncology, Bursa Uludağ University Faculty of Medicine, between January 2010 and January 2025. Clinical and survival outcomes, which had been routinely recorded in the institutional electronic medical record system during standard oncological follow-up, were retrospectively reviewed up to a predefined data cut-off of 17 November 2025. Eligible patients were adults with a documented diagnosis of mRCC who initiated first-line systemic therapy at our institution and had sufficient pretreatment clinical, laboratory, treatment, and follow-up data for analysis. Patients without documented metastatic disease during the study period, those whose oncological follow-up was conducted primarily at another center, and those with insufficient baseline or outcome data were excluded. Patients who had previously received systemic treatment for metastatic disease were not eligible.

### 2.2. Clinical Data Collection

Demographic, clinical, pathological, treatment-related, and laboratory data were retrospectively extracted from electronic health records. Recorded variables included age, sex, Karnofsky Performance Status (KPS), relevant comorbidities, histological subtype, metastatic disease characteristics, and systemic anticancer treatments administered during the course of the disease. KPS was assessed during routine clinical care by the treating medical oncologist, and histological subtype was obtained from pathology reports issued by specialist pathologists. Baseline clinical and laboratory parameters were used to determine the IMDC and MSKCC risk groups. The IMDC model comprised six adverse factors: KPS < 80%, hemoglobin below the lower limit of normal, corrected serum calcium above the upper limit of normal, neutrophil and platelet counts above their respective upper limits of normal, and an interval of <1 year from diagnosis to systemic treatment initiation. Comorbidity was entered consistently as a binary variable—any documented comorbidity versus none—in both the conventional and neural-network analyses. The MSKCC classification was calculated according to the updated 2002 five-factor model [8], which includes the diagnosis-to-treatment interval rather than prior nephrectomy, comprising Karnofsky Performance Status < 80%, hemoglobin below the lower limit of normal, corrected serum calcium above the upper limit of normal, lactate dehydrogenase > 1.5 times the upper limit of normal, and an interval of <1 year from diagnosis to initiation of systemic treatment. In this updated model, the diagnosis-to-treatment interval replaced prior nephrectomy as a risk factor; therefore, nephrectomy was not included as a component of the MSKCC score entered into the Cox or neural-network analyses. Patients with 0, 1–2, or ≥3 adverse factors were classified as favorable-, intermediate-, or poor-risk, respectively. Treatment regimens, treatment sequences, disease progression, and survival data were obtained from longitudinal oncology follow-up records.

Comorbidity status was categorized dichotomously according to whether at least one chronic medical condition was documented in the medical record. The conditions considered for this classification were diabetes mellitus, hypertension, cardiovascular disease, and chronic kidney disease. To avoid model overparameterization and estimates based on small subgroups, these conditions were not entered individually into the primary survival analyses.

### 2.3. Laboratory Assessment and Calculation of Inflammation- and Nutrition-Based Indices

**Figure 1:**
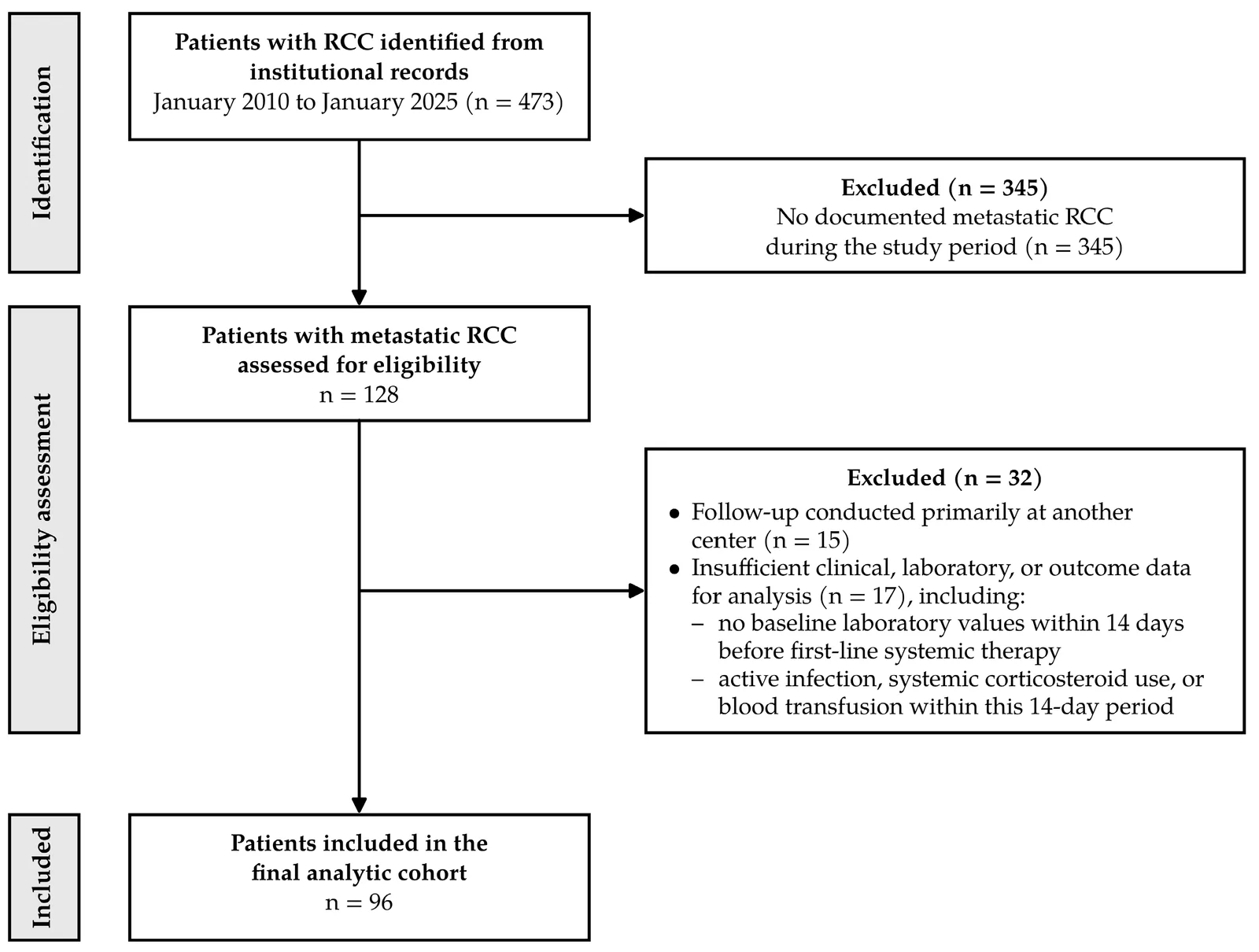
Flow diagram of patient identification, eligibility assessment, and inclusion in the final analytic cohort.

Hematological and biochemical parameters were obtained from the most recent routine laboratory examination performed within 14 days before the initiation of first-line systemic therapy for metastatic disease. Patients without a valid baseline laboratory examination in this window, including those with documented active infection, systemic corticosteroid use, or blood transfusion during this period, were classified as having insufficient laboratory data and are included in the corresponding exclusion category of the patient flow diagram (Figure 1). These baseline measurements were used for clinical risk assessment and for the calculation of the inflammatory and nutritional indices. Hemoglobin concentration, serum albumin level, and absolute neutrophil, lymphocyte, monocyte, and platelet counts were recorded. PIV and HALP were calculated using baseline hematological and biochemical parameters. PIV was calculated as absolute neutrophil count (×10^9^/L) × platelet count (×10^9^/L) × monocyte count (×10^9^/L)/absolute lymphocyte count (×10^9^/L). HALP score was calculated as hemoglobin (g/L) × serum albumin (g/L) × absolute lymphocyte count (×10^9^/L)/platelet count (×10^9^/L). Hemoglobin and albumin values recorded in g/dL were converted to g/L before calculation. MPV was categorized according to the institutional laboratory reference range as low (<6.9 fL), normal (6.9–10.6 fL), or high (>10.6 fL) and was evaluated exploratorily as a routinely available marker of platelet activation.

### 2.4. Outcome Definitions and Follow-Up

Clinical outcomes were determined through a detailed review of longitudinal electronic medical records, including oncology follow-up visits, radiological assessments, hospitalization records, and treatment documentation. PFS was defined as the time from the initiation of first-line systemic therapy for metastatic disease to the occurrence of either documented disease progression or death from any cause, whichever occurred first. Both progression and death were therefore considered PFS events. Patients who had experienced neither progression nor death by the data cut-off date were censored on 17 November 2025. OS was defined as the time from the initiation of first-line systemic therapy for metastatic disease to death from any cause. Death from any cause was considered an OS event. Patients who were alive at the end of the observation period were censored on the data cut-off date of 17 November 2025. Disease progression was investigator-assessed retrospectively from serial radiological examinations and contemporaneous oncology records, without blinded central imaging review. Imaging assessments were generally performed at approximately 3-month intervals, with additional imaging when clinically indicated. Death without previously documented progression was counted as a PFS event and occurred in 22 patients (22.9% of the cohort).

### 2.5. Ethical Considerations

This study was designed as a retrospective analysis of pre-existing, routinely collected clinical data; patient diagnosis, treatment, and follow-up occurred as part of standard clinical care, independent of and prior to this research protocol. The retrospective review and extraction of these records for research purposes was conducted following approval by the Bursa Uludağ University Faculty of Medicine Ethics Committee (Approval No: 2026/1/1-24, dated 7 January 2026), in accordance with the ethical principles of the Declaration of Helsinki.

### 2.6. Statistical Analysis

The distribution of continuous variables was assessed for normality using the Shapiro–Wilk test. Normally distributed continuous variables were summarized as mean ± standard deviation, whereas non-normally distributed variables were presented as median, minimum, and maximum values. Categorical variables were summarized as frequencies and percentages. The extent of missing data was reported for each variable. No imputation was performed for the conventional survival analyses. Kaplan–Meier analyses were based on the available observations for each variable, whereas Cox regression models used complete-case analysis with listwise exclusion of patients with missing data in any variable entered into the respective initial model. Survival outcomes were estimated using the Kaplan–Meier method, and survival distributions between groups were compared using the log-rank test. Median survival times were reported together with their corresponding 95% confidence intervals (CIs). Median follow-up was estimated using the reverse Kaplan–Meier method. Cox proportional hazards regression analyses were performed to evaluate factors associated with PFS and OS while accounting for the simultaneous effects of potential prognostic variables. Multivariable models were constructed using a backward stepwise procedure based on the likelihood ratio criterion. Effect estimates from the Cox models were reported as hazard ratios (HRs) with 95% CIs. All statistical analyses were performed using IBM SPSS Statistics, version 27.0 (IBM Corp., Armonk, NY, USA). All statistical tests were two-sided, and a *p* value < 0.05 was considered statistically significant. Tables and Kaplan–Meier figures were generated in R (version 4.5.2; R Core Team, Vienna, Austria) using the survival (version 3.8.12) and ggplot2 (version 4.0.3) packages. The proportional-hazards assumption was evaluated using extended Cox regression models incorporating covariate-by-log(time) interaction terms. A statistically significant time-dependent interaction was considered evidence of violation of the proportional hazards assumption. Cox analyses were performed using IBM SPSS Statistics version 27.0, whereas the software and packages used for the neural-network analyses are reported in Section 2.7.

### 2.7. Neural-Network Survival Analysis

As an exploratory analysis, a dual-head feed-forward neural network (multilayer perceptron) was developed to predict individual overall survival from baseline variables. Thirteen candidate predictors were considered: age (continuous), sex, any comorbidity, nephrectomy status, the IMDC and MSKCC factor counts, log-transformed PIV, HALP, mean platelet volume (MPV, continuous), clear-cell histology, and bone, lung and brain metastasis. For neural-network modelling, nephrectomy was encoded as a binary ever-nephrectomy variable. The detailed clinical timing categories were reported descriptively in the clinical analyses and were not entered as separate neural-network predictors. In a sensitivity analysis, the six patients who underwent nephrectomy after the start of first-line therapy were coded as not having undergone nephrectomy at baseline, and the complete nested analysis was repeated (Supplementary Methods S1.6; Table S5). KPS and the diagnosis-to-treatment interval were not entered separately because they are components of the IMDC and MSKCC scores, and treatment type was not considered because it reflects a treatment decision rather than a patient characteristic and was highly imbalanced (86 patients treated with TKI monotherapy vs. 10 with ICI-based regimens). The IMDC and MSKCC factor counts were entered as ordinal inputs; missing values (histology, *n* = 9; bone metastasis, *n* = 1) were imputed within each training fold. In one patient with missing LDH, the MSKCC factor count was derived from the remaining MSKCC components (LDH assumed normal), because the network requires complete inputs; in the conventional analyses, this patient was treated as missing for MSKCC. The network had a shared trunk of two fully connected layers (32 and 16 units) and two output heads: a risk head trained with the Cox partial likelihood and a time head predicting log survival time with a censoring-aware Huber loss [19,20]. Because the number of events (*n* = 57) was small relative to the number of candidate predictors (13) and network parameters (about 1000), permutation-importance feature screening was used to reduce the risk of overfitting [21,22,23]. Screening and hyperparameter tuning were nested within stratified 5-fold cross-validation repeated 10 times (50 outer folds) [24], and the network was also trained without screening as a sensitivity analysis. Details of the architecture, training, hyperparameter grid, preprocessing and performance measures are given in Supplementary Methods S1. In the primary analyses, nephrectomy was defined as ever-nephrectomy, reflecting whether a patient underwent nephrectomy at any time during the documented disease course. Because six patients underwent nephrectomy after initiation of first-line systemic therapy, a post hoc sensitivity analysis was performed in which nephrectomy status was redefined at the initiation of first-line treatment. These six patients were classified as having no nephrectomy at baseline, resulting in 63 patients with and 33 patients without nephrectomy at treatment initiation. Kaplan–Meier and multivariable Cox analyses were repeated using this baseline definition. In addition, the complete nested neural-network analysis, including feature screening, hyperparameter tuning, all benchmark models, and the leave-one-feature-out analysis, was repeated using the same recoding across the identical 50 outer folds. On the identical outer splits, the network was compared with a Kaplan–Meier null model, Cox models containing the IMDC or MSKCC risk group alone or the IMDC risk group plus nephrectomy status, a standard Cox model with the network-selected predictors, a ridge-penalized Cox model with all candidates, an elastic-net (LASSO-type) Cox model and a random survival forest [25,26]. Discrimination was assessed with Harrell’s C-index, reported as the fold-wise mean (SD) and as a pooled estimate with bootstrap 95% CIs [27,28]. For the network, the accuracy of individual survival probabilities was assessed with IPCW Brier scores at 6, 12, 24 and 36 months and the integrated Brier score against a Kaplan–Meier reference [29,30], and calibration with calibration plots, calibration-in-the-large and a calibration slope [31]. The mean and median absolute errors (MAEs, MedAEs) of the predicted survival time were computed in patients who died, and variable contributions were explored with leave-one-feature-out (LOFO) analysis. Analyses were performed in Python 3.9.13 with PyTorch 2.8.0, lifelines 0.30.0, scikit-survival 0.23.1 and scikit-learn 1.5.2 [32,33,34] and are reported according to TRIPOD+AI [35]; the analysis code is available on Zenodo at https://doi.org/10.5281/zenodo.23515898.

### 2.8. Use of Artificial Intelligence Tools

The AI-based language-editing tool ChatGPT (GPT-5; OpenAI) was used during manuscript preparation to improve the clarity and readability of the author-drafted text. This tool was not used for study conceptualization, the design or execution of the statistical or neural-network analyses, data collection or curation, interpretation of results, formulation of conclusions, or the generation of any figures or images.

## 3. Results

Of 473 patients with RCC identified from institutional records between January 2010 and January 2025, 345 had no documented metastatic RCC during the study period. Of the remaining 128 patients assessed for eligibility, 32 were excluded because follow-up was conducted primarily at another center (*n* = 15) or because clinical, laboratory, or outcome data were insufficient for analysis (*n* = 17). Thus, 96 patients were included in the final analytic cohort (Figure 1). Of the 96 included patients, 72 (75.0%) were male and 24 (25.0%) were female. The mean age was 62.1 years (SD, 10.9; range, 31–81 years) (Table 1).

**Figure 2:**
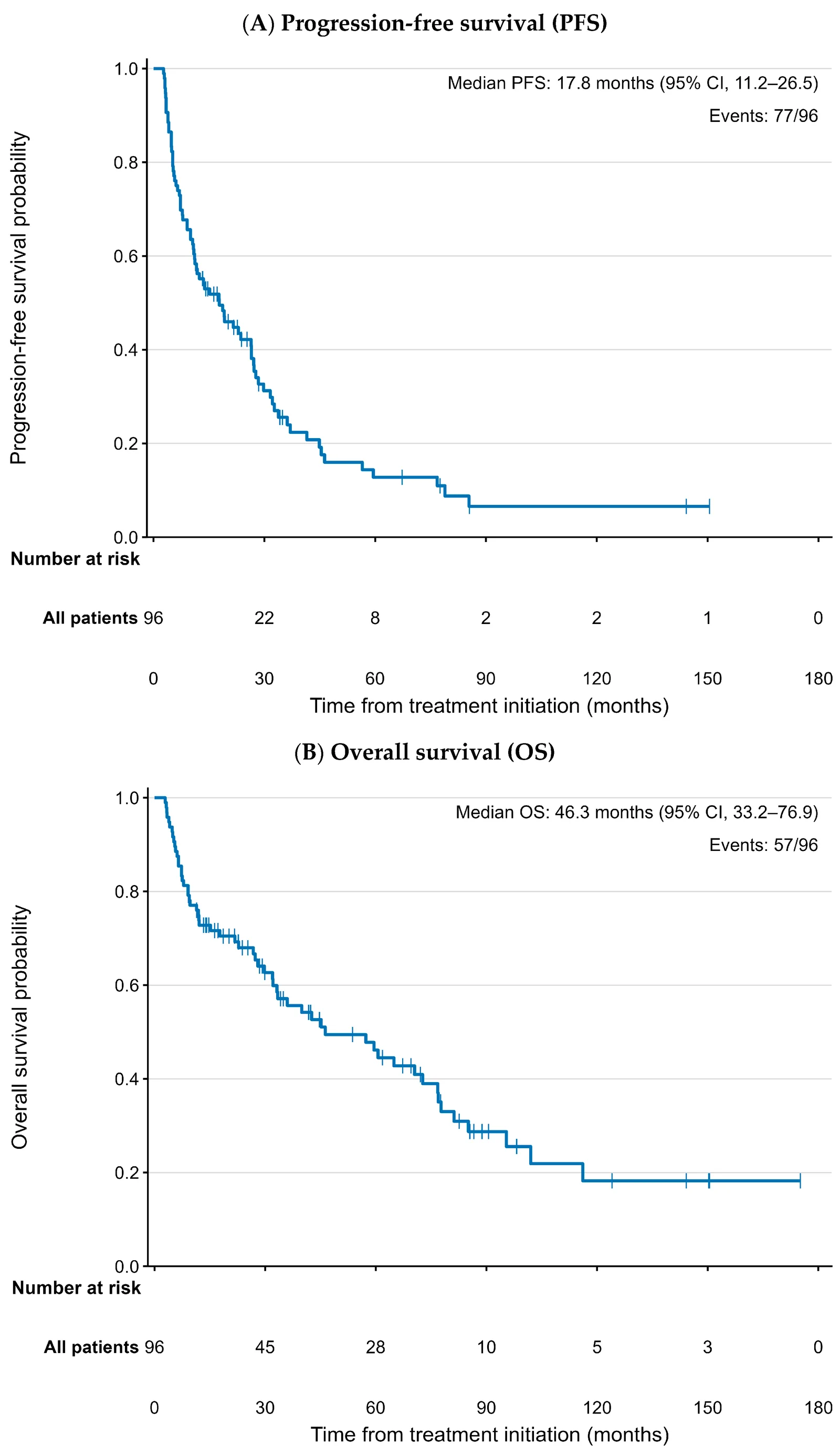
Kaplan–Meier survival curves for the study cohort. (**A**) Progression-free survival (PFS); (**B**) overall survival (OS). Tick marks indicate censored observations; numbers at risk are shown beneath the x-axis.

For the entire cohort, median PFS was 17.8 months (95% CI, 8.86–26.74) and median OS was 46.3 months (95% CI, 21.84–70.76) (Figure 2). At the data cut-off, 77 PFS events and 57 deaths had occurred. The median follow-up estimated using the reverse Kaplan–Meier method was 82.6 months. Among the 69 patients who underwent nephrectomy, 41 had undergone prior nephrectomy followed by metachronous metastatic relapse, 22 underwent nephrectomy before first-line systemic therapy in the setting of synchronous metastatic disease, five underwent deferred nephrectomy after systemic treatment initiation, and one underwent palliative nephrectomy. Outcomes were not analysed separately across these subgroups because of their small and markedly unequal sizes and because nephrectomy indications and treatment approaches evolved during the study period. These categories are therefore presented descriptively.

### 3.1. Progression-Free Survival Analyses

In analyses according to demographic and clinical characteristics, PFS did not differ significantly by sex (*p* = 0.289), age group (<65 vs. ≥65 years, *p* = 0.949), smoking status (*p* = 0.698), or alcohol consumption (*p* = 0.753). Although the sex-based comparison was not statistically significant, median PFS was numerically shorter in women than in men (7.3 vs. 19.1 months) (Table S1).

In contrast, Karnofsky Performance Status (KPS) was significantly associated with PFS. Patients with a KPS >80 had a longer median PFS than those with a KPS ≤80 (23.0 vs. 7.9 months, *p* = 0.025) (Table S1).

The presence of comorbidity as an overall variable was not associated with PFS (*p* = 0.840). Regarding disease-related characteristics, no significant differences in PFS were observed according to histological subtype (*p* = 0.540), bone metastasis (*p* = 0.373), lung metastasis (*p* = 0.871), or brain metastasis (*p* = 0.146) (Table S1).

International Metastatic Renal Cell Carcinoma Database Consortium (IMDC) risk groups differed significantly in PFS (log-rank *p* = 0.027). Median PFS was 26.5 months in the favorable-risk group, 17.7 months in the intermediate-risk group, and 7.9 months in the poor-risk group. In pairwise comparisons, patients in the poor-risk group had significantly shorter PFS than those in the favorable-risk group (*p* = 0.013) and the intermediate-risk group (*p* = 0.031), whereas no significant difference was observed between the favorable- and intermediate-risk groups (*p* = 0.361) (Table S1; Figure S1). In contrast, PFS did not differ significantly across Memorial Sloan Kettering Cancer Center (MSKCC) risk groups (log-rank *p* = 0.284), although median PFS showed a numerical gradient of 26.5, 13.8, and 7.0 months in the favorable-, intermediate-, and poor-risk groups, respectively (Table S1; Figure S2).

With respect to treatment-related factors, PFS did not differ significantly between patients treated with tyrosine kinase inhibitor (TKI)-based therapy and those receiving immunotherapy or combination regimens (*p* = 0.357). No significant difference was observed among individual TKI agents (*p* = 0.909) or according to the interval between diagnosis and treatment (<1 vs. ≥1 year, *p* = 0.208). Patients who underwent nephrectomy had a significantly longer median PFS than those who did not (26.4 vs. 10.0 months; *p* = 0.002) (Table S1; Figure S3).

Among the evaluated inflammatory and nutritional biomarkers, no significant differences in PFS were observed according to median-based PIV (≤325.97 vs. >325.97, *p* = 0.330) and HALP (>34.68 vs. ≤34.68, *p* = 0.167) categories. Similarly, PFS did not differ significantly according to mean platelet volume categories (*p* = 0.388; Table S1)

### 3.2. Overall Survival Analyses

In analyses according to demographic and clinical characteristics, OS did not differ significantly by sex (*p* = 0.232), age group (<65 vs. ≥65 years, *p* = 0.954), smoking status (*p* = 0.128), or alcohol consumption (*p* = 0.580). In contrast, KPS was significantly associated with OS. Patients with a KPS >80 had a substantially longer median OS than those with a KPS ≤80 (70.5 vs. 12.0 months, *p* < 0.001) (Table S2).

The presence of comorbidity as an overall variable was not associated with OS (*p* = 0.839). Regarding disease-related characteristics, OS did not differ significantly according to histological subtype (*p* = 0.208) or lung metastasis (*p* = 0.161). In contrast, the presence of bone metastasis was associated with shorter median OS (39.9 vs. 64.9 months in patients with and without bone metastasis, respectively; *p* = 0.042). Similarly, patients with brain metastasis had a markedly shorter median OS than those without brain metastasis (11.4 vs. 59.5 months, *p* = 0.034) (Table S2).

IMDC risk groups differed significantly in OS (*p* = 0.001). Median OS was 95.4, 45.2, and 9.1 months in the favorable-, intermediate-, and poor-risk groups, respectively. In pairwise comparisons, OS was significantly shorter in the poor-risk group than in both the favorable-risk group (*p* = 0.003) and the intermediate-risk group (*p* = 0.006), whereas the difference between the favorable- and intermediate-risk groups was not statistically significant (*p* = 0.061) (Table S2; Figure S4). MSKCC risk groups were also significantly associated with OS (log-rank *p* = 0.018), with median OS values of 95.4, 42.6, and 9.6 months in the favorable-, intermediate-, and poor-risk groups, respectively. Pairwise comparisons showed a significant difference between the favorable- and intermediate-risk groups (*p* = 0.043) and between the favorable- and poor-risk groups (*p* = 0.037), whereas the difference between the intermediate- and poor-risk groups was not statistically significant (*p* = 0.073) (Table S2; Figure S5).

With respect to treatment-related factors, OS did not differ significantly between patients receiving TKI-based therapy and those treated with immunotherapy or combination regimens (*p* = 0.888), or among individual TKI agents (*p* = 0.527). In contrast, patients with a diagnosis-to-treatment interval of ≥1 year had a longer median OS than those with an interval of <1 year (76.8 vs. 33.2 months, *p* = 0.043). Nephrectomy was associated with significantly longer OS, with a median OS of 76.8 months in patients who underwent nephrectomy compared with 11.9 months in those who did not (*p* < 0.001) (Table S2; Figure S6).

Among the evaluated inflammatory and nutritional biomarkers, lower PIV was associated with longer median OS (64.9 vs. 33.4 months for PIV ≤325.97 vs. >325.97, respectively; *p* = 0.032). Patients with HALP >34.68 also had longer median OS than those with HALP ≤34.68 (81.2 vs. 36.0 months, *p* = 0.040). OS did not differ significantly according to MPV categories (*p* = 0.604) (Table S2).

### 3.3. Multivariable Cox Regression Analysis for Progression-Free Survival

For the IMDC-based Cox regression analysis, variables were selected according to both statistical associations observed in the unadjusted analyses and their established clinical relevance. Age, nephrectomy status, and IMDC risk classification were entered into the initial model. IMDC risk classification and nephrectomy status were included because of their associations with PFS in the unadjusted analyses, whereas age was included as a continuous covariate because of its clinical relevance. KPS was not entered separately to avoid overlap with performance-status information already incorporated within the composite IMDC classification. Following backward stepwise selection, age was removed first. The overall effect of IMDC subsequently did not meet the prespecified retention criterion (*p* = 0.111) and was removed from the model. In the final step, nephrectomy status was the only retained variable. The final model was statistically significant (*p* = 0.002). Patients who had not undergone nephrectomy had a 2.25-fold higher hazard of progression or death compared with those who had undergone nephrectomy (HR, 2.25; 95% CI, 1.32–3.81; *p* = 0.003) (Table 2).

For the MSKCC-based analysis, age, nephrectomy status, and MSKCC risk classification were entered into the initial Cox regression model. Although MSKCC risk classification was not significantly associated with PFS in the unadjusted analysis, it was evaluated in a separate multivariable model because of its established prognostic relevance in metastatic renal cell carcinoma and its significant association with OS in the present cohort. IMDC and MSKCC classifications were evaluated in separate models to avoid simultaneous inclusion of overlapping composite prognostic scores. Following backward stepwise selection, age was removed first. MSKCC risk classification subsequently did not meet the prespecified retention criterion (*p* = 0.454) and was removed from the model. In the final step, nephrectomy status was the only retained variable. The final model was statistically significant (*p* = 0.002). Patients who had not undergone nephrectomy had a 2.25-fold higher hazard of progression or death compared with those who had undergone nephrectomy (HR, 2.25; 95% CI, 1.32–3.84; *p* = 0.003) (Table 2).

To evaluate clinical prognostic factors independently of the composite IMDC and MSKCC classifications, a separate score-free Cox model was constructed including age, nephrectomy status, and KPS. Following backward stepwise selection, age and KPS were removed, leaving nephrectomy status as the only variable retained in the final model. Patients who had not undergone nephrectomy had a 2.24-fold higher hazard of progression or death compared with those who had undergone nephrectomy (HR, 2.24; 95% CI, 1.32–3.81; *p* = 0.003) (Table 2).

### 3.4. Multivariable Cox Regression Analysis for Overall Survival

For the IMDC-based multivariable analysis, age, IMDC risk classification, bone metastasis, brain metastasis, nephrectomy status, PIV, and HALP were entered into the initial Cox regression model. KPS and the diagnosis-to-treatment interval were not entered separately because both are incorporated into the IMDC prognostic classification, thereby avoiding redundancy with the composite risk score. Following backward stepwise selection, HALP, brain metastasis, PIV, and bone metastasis were sequentially removed, whereas age, IMDC risk classification, and nephrectomy status were retained in the final model. IMDC risk classification remained significantly associated with OS overall (*p* = 0.024). Using the poor-risk group as the reference, patients in the favorable-risk group had a 73% lower hazard of death (HR, 0.27; 95% CI, 0.09–0.79; *p* = 0.016), while those in the intermediate-risk group had a 55% lower hazard of death (HR, 0.45; 95% CI, 0.23–0.87; *p* = 0.017). Age was retained in the final model but did not reach conventional statistical significance (HR, 1.03; 95% CI, 1.00–1.05; *p* = 0.060). Patients who had not undergone nephrectomy had a 4.48-fold higher hazard of death than those who had undergone nephrectomy (HR, 4.48; 95% CI, 2.33–8.60; *p* < 0.001). The final model was statistically significant (*p* < 0.001) (Table 3).

For the MSKCC-based multivariable analysis, age, MSKCC risk classification, bone metastasis, brain metastasis, nephrectomy status, PIV, and HALP were entered into the initial Cox regression model. Following backward stepwise selection, HALP, brain metastasis, and bone metastasis were sequentially removed, whereas age, PIV, MSKCC risk classification, and nephrectomy status were retained in the final model. MSKCC risk classification remained significantly associated with OS overall (*p* = 0.032). Using the poor-risk group as the reference, patients in the favorable-risk group had a 74% lower hazard of death (HR, 0.26; 95% CI, 0.09–0.78; *p* = 0.016), while those in the intermediate-risk group had a 60% lower hazard of death (HR, 0.40; 95% CI, 0.18–0.89; *p* = 0.024). Age (HR, 1.02; 95% CI, 1.00–1.05; *p* = 0.103) and PIV (HR, 1.66; 95% CI, 0.91–3.04; *p* = 0.101) were retained in the final model but did not reach conventional statistical significance. Patients who had not undergone nephrectomy had a 4.66-fold higher hazard of death compared with those who had undergone nephrectomy (HR, 4.66; 95% CI, 2.37–9.16; *p* < 0.001). The final model was statistically significant (*p* < 0.001) (Table 3).

To evaluate prognostic factors independently of the composite IMDC and MSKCC classifications, a separate score-free Cox model was constructed including age, bone metastasis, brain metastasis, nephrectomy status, HALP, PIV, diagnosis-to-treatment interval, and KPS. Following backward stepwise selection, brain metastasis, bone metastasis, HALP, diagnosis-to-treatment interval, and age were removed, whereas nephrectomy status, PIV, and KPS were retained in the final model. Patients who had not undergone nephrectomy had a 3.79-fold higher hazard of death compared with those who had undergone nephrectomy (HR, 3.79; 95% CI, 1.94–7.42; *p* < 0.001). Patients with a KPS ≤ 80 had a 2.31-fold higher hazard of death than those with a KPS > 80 (HR, 2.31; 95% CI, 1.25–4.28; *p* = 0.008). Higher PIV was also retained in the final model, although its association with OS did not reach statistical significance (HR, 1.64; 95% CI, 0.93–2.92; *p* = 0.090). The final model was statistically significant (*p* < 0.001) (Table 3). No significant covariate-by-log(time) interactions were observed in either the PFS or OS Cox models (all *p* > 0.05), providing no evidence of violation of the proportional-hazards assumption. In a sensitivity analysis defining nephrectomy status strictly at the initiation of first-line systemic therapy, the six patients who underwent post-treatment nephrectomy were classified as having no nephrectomy at baseline. The association remained directionally consistent, although the effect estimates were attenuated. Absence of nephrectomy at baseline remained associated with shorter PFS across the Cox models (HR range, 1.66–1.82; *p* = 0.018–0.050) and with shorter OS (HR range, 2.26–2.78; all *p* ≤ 0.009). Unadjusted median PFS was 10.9 versus 23.0 months (*p* = 0.015), and median OS was 17.7 versus 72.7 months (*p* < 0.001), for patients without versus with nephrectomy at baseline, respectively (Table S6).

### 3.5. Exploratory Neural-Network Survival Analysis

**Figure 3:**
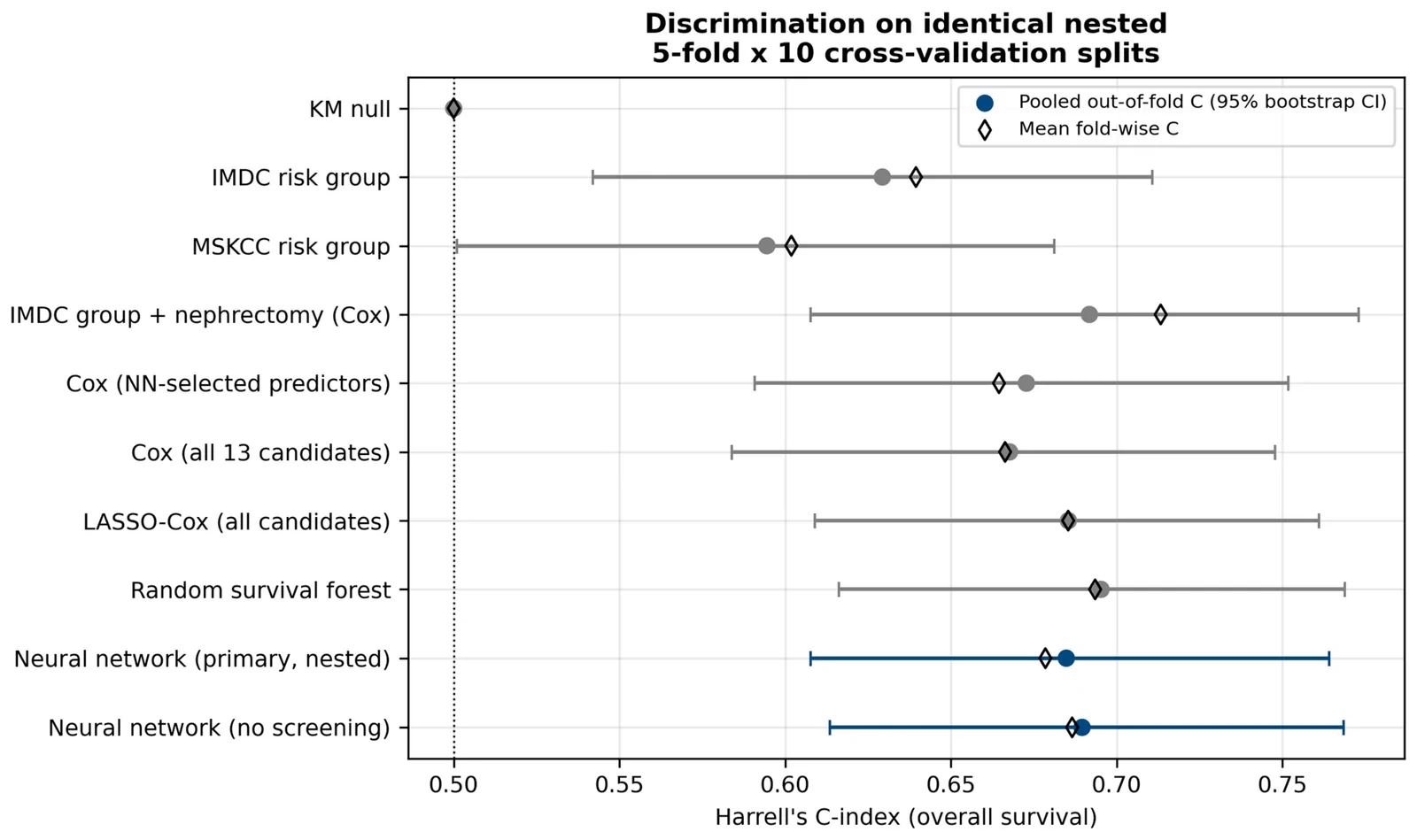
Discrimination of the neural network and comparator models for overall survival on identical nested cross-validation splits. Filled circles show the pooled out-of-fold Harrell’s C-index with 95% bootstrap confidence intervals; open diamonds show the mean of the 50 fold-wise C-indices. The dotted line marks C = 0.5 (no discrimination).

**Figure 4:**
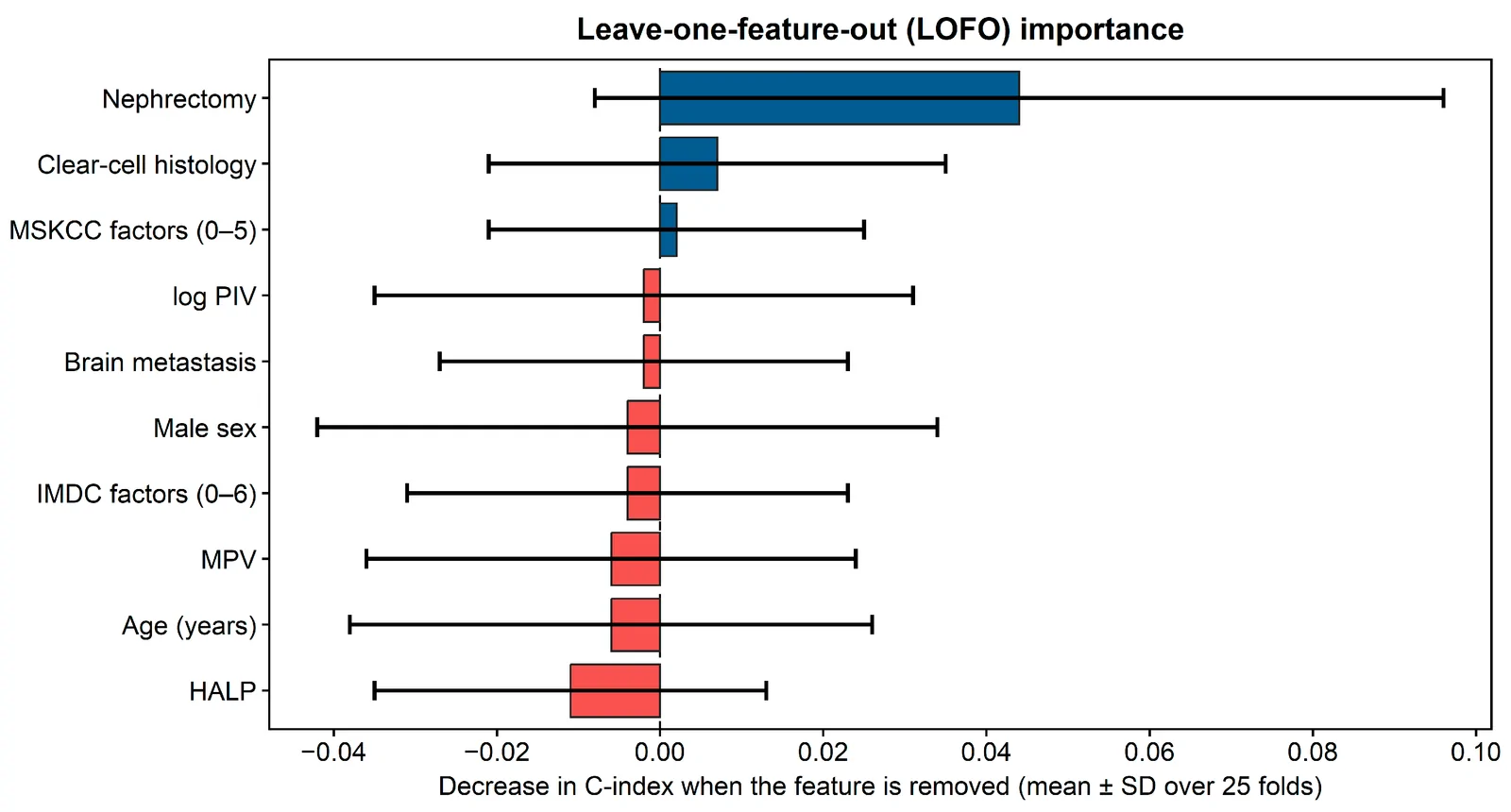
Leave-one-feature-out (LOFO) analysis of the full-cohort neural-network model. Bars show the mean decrease in validation C-index when each predictor is removed, and error bars the SD across 25 paired folds (5 × 5-fold cross-validation). Positive values indicate that the predictor contributed to discrimination; for all predictors the SD exceeds the mean.

In the nested evaluation, the network achieved a pooled C-index of 0.685 (95% CI, 0.608–0.764; fold-wise mean, 0.679 [SD, 0.084]), comparable to that of the Cox model with IMDC risk group and nephrectomy status (0.692) and the random survival forest (0.695), numerically higher than that of the IMDC risk group alone (0.629; ΔC, 0.055; 95% CI, −0.011 to 0.123) and higher than that of the MSKCC risk group alone (0.594; ΔC, 0.090; 95% CI, 0.003 to 0.175) (Table 4; Figure 3). Without screening, the C-index (0.690) and integrated Brier score (0.189) were similar and the calibration slope was comparable (0.52 vs. 0.54). Screening retained on average 10.0 of the 13 candidates, and only nephrectomy status was retained in every outer fold, whereas the other candidates were retained in 50–94% of folds (Figure S7; Table S3). In the LOFO analysis, removal of nephrectomy status produced by far the largest decrease in C-index (ΔC, 0.044 ± 0.052), whereas no other predictor changed the C-index by more than 0.011 (Figure 4).

**Figure 5:**
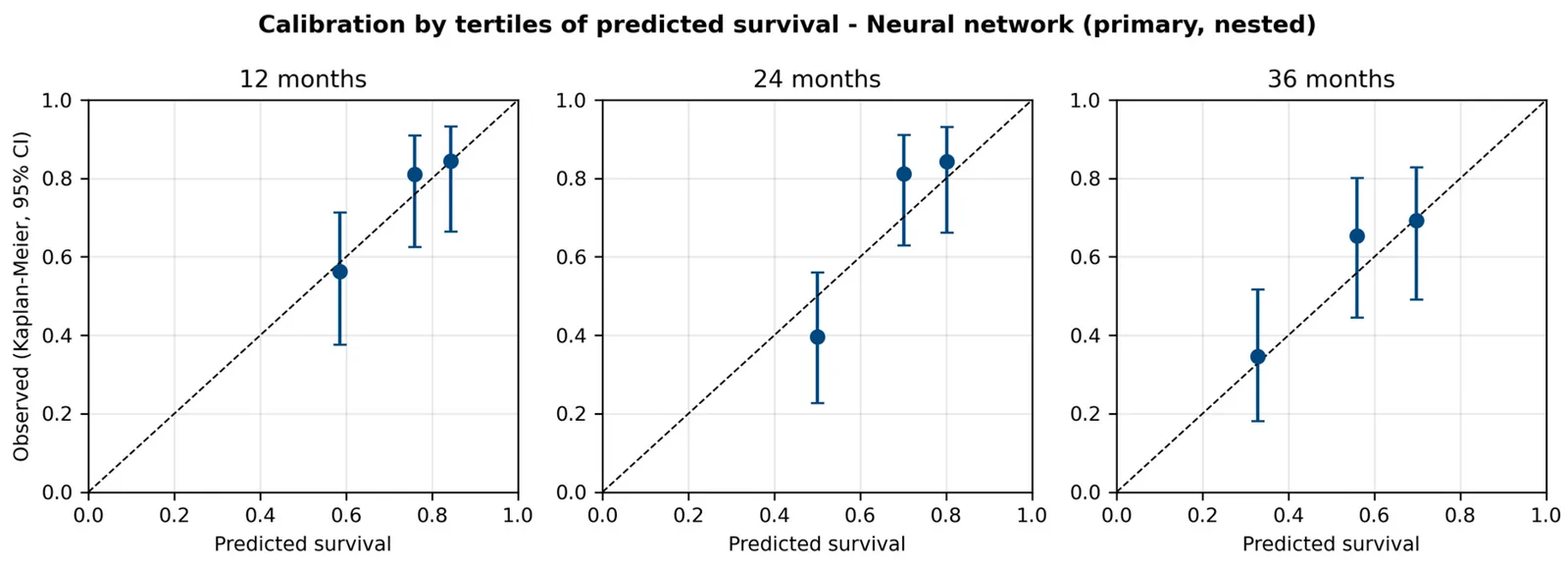
Calibration of the neural network at 12, 24 and 36 months. Mean nested out-of-fold predicted survival versus observed Kaplan–Meier survival (95% CI) within tertiles of predicted survival; the dashed line indicates perfect calibration.

The Brier scores of the network were lower than those of the Kaplan–Meier null model at all evaluated time points. Its integrated Brier score was 0.189, compared with 0.164 for the Cox model with IMDC risk group and nephrectomy status and 0.183 for the random survival forest, whose calibration slopes were 0.79 and 1.04, respectively (Table 5; Figure S8). Calibration-in-the-large was good (Table 5), but the calibration slope of 0.54 indicated overly extreme risk predictions; in the tertile-based calibration plots (Figure 5), observed and predicted survival agreed within the 95% confidence intervals at all three time points, although such grouped plots have limited ability to detect this type of miscalibration. Among patients who died, the MAE and MedAE of the predicted survival time were 23.9 and 20.1 months, respectively; because censored patients cannot be scored, these values are biased toward shorter survival. In the sensitivity analysis in which the six patients with nephrectomy after the start of first-line therapy were coded as not having undergone nephrectomy at baseline, discrimination decreased slightly for all models, and the network (C-index, 0.677; 95% CI, 0.601–0.758) remained comparable to the Cox model with IMDC risk group and nephrectomy status (0.672; 95% CI, 0.585–0.760; ΔC, 0.005; 95% CI, −0.056 to 0.062) and the random survival forest (0.672). Nephrectomy status was still retained in every outer fold and still made the largest LOFO contribution, although this contribution was smaller (ΔC, 0.024 ± 0.048) (Table S5).

## 4. Discussion

In this single-center real-world cohort of patients with mRCC, conventional survival analyses confirmed the prognostic relevance of established clinical risk stratification while identifying nephrectomy as one of the most prominent clinical variables associated with outcome. Median PFS and OS were 17.8 and 46.3 months, respectively, and substantial survival heterogeneity was observed according to performance status, IMDC and MSKCC risk classification, nephrectomy status, metastatic sites, and systemic inflammatory–nutritional indices, particularly for OS. A neural-network model showed discrimination comparable to conventional Cox-based models, but its individualized survival-probability estimates had greater prediction error than those of the two-variable Cox model and were poorly calibrated in slope. Its survival-time estimates also had limited accuracy; therefore, all model-derived estimates should be regarded as exploratory until recalibrated and externally validated. Age and sex did not significantly influence survival in our cohort. The numerically shorter PFS observed in women may partly reflect their lower proportions of favorable-risk disease according to the IMDC and MSKCC classifications and the greater representation of non-clear-cell histology. Although sex was retained during neural-network feature selection, its contribution in the leave-one-feature-out analysis was negligible; therefore, the small female subgroup and the non-significant conventional comparison preclude interpretation of sex as an independent prognostic factor. In a pooled analysis of 4736 patients with metastatic RCC treated in phase II and III trials, OS was likewise similar across age groups and between sexes; however, younger patients (<50 years) had a shorter median PFS than those aged 50–70 years [36]. This finding suggests that the relationship between chronological age and outcome in mRCC may not be linear and may vary according to the survival endpoint and treatment context.

The demographic profile of our cohort was broadly comparable with that of contemporary phase III mRCC trials. The mean age was 62.1 years and 75% of patients were male, closely resembling the median ages of approximately 61–64 years and male proportions of 71–77% reported in KEYNOTE-426, CLEAR and CheckMate 214 [3,37,38]. Only a small proportion received immune checkpoint inhibitor-based therapy, reflecting the long study period and predominance of the TKI era. Approximately 19.8% of patients had non-clear-cell histology, resulting in a more histologically heterogeneous population than those included in pivotal phase III trials. Accordingly, the survival estimates observed in our cohort should not be interpreted as evidence of superior treatment efficacy. Selection effects, treatment-era heterogeneity, subsequent therapies, differences in follow-up, and the retrospective ascertainment of progression may all have contributed to the observed outcomes and limit direct comparison with prospective randomized trials.

Notably, receipt of an immune checkpoint inhibitor-based regimen was not associated with significantly different survival in our cohort. Median PFS was 15.2 months with TKI therapy and 21.6 months with ICI-based or combination therapy (*p* = 0.357), while median OS was 57.3 months (95% CI, 32.90–81.70) and 28.0 months (95% CI, not estimable), respectively (*p* = 0.888). The median OS estimate for the ICI-based or combination-therapy subgroup should be interpreted cautiously because it was based on only 10 patients and a limited number of deaths. Furthermore, these patients were generally treated more recently and consequently had shorter potential follow-up, which may have influenced the apparently shorter OS estimate. A similar absence of a statistically significant OS difference among treatment groups was observed in a large US real-world study of 1538 patients, in which median OS was not reached with pembrolizumab–axitinib, 27.6 months with nivolumab–ipilimumab, and 26.9 months with TKI monotherapy (*p* = 0.237) [39]. This should not be interpreted as evidence of therapeutic equivalence, since randomized trials have demonstrated durable PFS and OS advantages for modern ICI-based combinations [37]. In our study, only 10 patients received ICI-based or combination therapy compared with 86 treated with TKI monotherapy, and the long study period introduced substantial treatment-era and follow-up heterogeneity; therefore, the absence of statistical significance is most plausibly explained by limited power and treatment-selection effects rather than lack of efficacy of immunotherapy.

Survival outcomes did not differ significantly according to the specific TKI used in our cohort. This finding is broadly compatible with the similar efficacy reported for pazopanib and sunitinib in the COMPARZ trial, whereas CABOSUN demonstrated a PFS advantage for cabozantinib over sunitinib in patients with intermediate- or poor-risk disease [40,41]. In our series, however, median OS could not be estimated for the cabozantinib subgroup, and the lack of an observed difference between individual TKIs should be interpreted in the context of small subgroup sizes and non-random treatment allocation.

Histologic subtype was not significantly associated with either PFS or OS in our cohort, despite the inclusion of a substantial non-clear-cell population. Similar findings have been reported in real-world series; in a recent Turkish multicenter cohort treated with sunitinib, median OS was numerically longer in clear-cell than in non-clear-cell RCC (34.6 vs. 22.8 months), but the difference was not statistically significant (*p* = 0.247) [42]. Given the biological heterogeneity of non-clear-cell RCC and the relatively small number of patients within individual histologic subtypes, the absence of a survival difference in our cohort should be interpreted cautiously.

Performance status was one of the strongest clinical prognostic factors in our cohort. Patients with KPS > 80 had substantially longer PFS and OS than those with KPS ≤ 80, and lower KPS remained independently associated with worse OS in the score-free multivariable model (HR, 2.31; *p* = 0.008). Similar findings have been reported in the immunotherapy era, where KPS < 80% was independently associated with poorer OS (HR, 3.42; *p* = 0.001) [43]. These findings reinforce the enduring prognostic value of functional status in mRCC, irrespective of treatment era.

The prognostic performance of the IMDC and MSKCC classifications differed according to the survival endpoint. IMDC risk groups significantly stratified both PFS and OS in the unadjusted analyses, whereas MSKCC risk classification was significantly associated with OS but not with PFS. However, PFS did not demonstrate a strictly monotonic gradient across worsening risk categories, because the favorable- and intermediate-risk IMDC groups had similar survival estimates and the corresponding MSKCC curves showed substantial overlap. In the multivariable PFS models, neither IMDC nor MSKCC remained in the final backward-selected models, with nephrectomy status emerging as the only retained variable. In contrast, both IMDC and MSKCC remained significantly associated with OS after multivariable adjustment, with lower hazards of death in the favorable- and intermediate-risk groups compared with the poor-risk group. These findings are consistent with contemporary evidence supporting the continued prognostic utility of established risk models, although their discriminatory performance may diminish with prolonged survival in the immunotherapy era [9].

Bone and brain metastases were associated with shorter OS in unadjusted analyses, whereas lung involvement was not significantly related to survival. However, neither bone nor brain metastasis retained an independent association with OS after multivariable adjustment, suggesting that their prognostic impact may be partly captured by other clinical factors and established risk classifications. The adverse survival pattern observed with brain metastases nevertheless remains consistent with contemporary IMDC data [44].

A longer interval between diagnosis and initiation of systemic therapy was associated with better OS in our cohort, with median OS of 76.8 months for patients with an interval ≥ 1 year versus 33.2 months for those treated within the first year. In the multicenter study by Heng et al., conducted in patients receiving VEGF-targeted agents, a diagnosis-to-treatment interval of <1 year was likewise identified as an adverse prognostic factor for OS [4]. This treatment context is broadly comparable with our cohort, in which TKI-based therapy predominated. However, the interval did not retain an independent association with OS in our score-free multivariable model, suggesting overlap with other indicators of disease aggressiveness and patient condition.

Nephrectomy status was associated with longer PFS and OS in our cohort. Larger IMDC observational studies reported an association between cytoreductive nephrectomy and longer OS after statistical adjustment; however, residual selection bias remained a concern, and our smaller heterogeneous cohort was not designed to estimate the causal effect of nephrectomy or to compare surgical strategies [45,46]. However, the nephrectomy group was clinically heterogeneous: 41 patients had undergone prior nephrectomy followed by metachronous metastatic relapse, 22 underwent nephrectomy before first-line systemic therapy in the setting of recorded synchronous metastatic disease, five underwent deferred nephrectomy after systemic treatment initiation, and one underwent palliative nephrectomy. Nephrectomy status may therefore reflect differences in disease biology, metastatic timing, tumour burden, performance status, treatment response, and clinical selection rather than an independent therapeutic effect. Outcomes were not compared across these subgroups because of their small and markedly unequal sizes and because nephrectomy indications and systemic treatment approaches evolved during the study period. In addition, classifying the six patients who underwent surgery after treatment initiation according to ever-nephrectomy status may have introduced immortal-time and responder-selection biases. Accordingly, our findings are not directly comparable with the selected populations evaluated in CARMENA, SURTIME, or ICI-era studies and should be interpreted as an observational association rather than evidence of a causal survival benefit [47,48,49].

Systemic inflammatory and nutritional indices showed model-dependent prognostic associations in our cohort. Lower PIV and higher HALP were associated with longer OS in unadjusted analyses, whereas neither marker was significantly associated with PFS. However, HALP did not retain an independent association after multivariable adjustment, and the effect of PIV was attenuated in the score-free model. Similar variability has been reported in TKI-treated populations. In a large cohort of 1001 patients receiving first-line TKIs, the association between HALP and OS lost statistical significance after adjustment for IMDC risk, while a recent first-line TKI study also found no significant association of PIV with either PFS or OS [50,51]. Together with positive findings reported in nivolumab-treated cohorts [10,11], these observations suggest that the prognostic contribution of inflammatory indices may depend on treatment setting, patient composition, and their overlap with established clinical risk factors.

Given the heterogeneous prognostic information provided by conventional risk scores and inflammatory–nutritional markers, we further explored whether these variables could be integrated within a nonlinear prediction framework. With feature screening and tuning nested within repeated cross-validation, the neural network showed moderate discrimination (C-index, 0.685), comparable to that of a parsimonious Cox model combining IMDC risk group and nephrectomy status and of a random survival forest, numerically higher than that of the IMDC risk group alone and higher than that of the MSKCC risk group alone. Most of this advantage over the risk scores appears to be attributable to nephrectomy status, which made by far the largest contribution in the LOFO analysis, whereas the contributions of PIV, HALP, sex and the other predictors were negligible; we therefore do not interpret the network as evidence of independent prognostic value for any single variable. The good performance of the two-variable Cox model reflects the dominant, and probably partly confounded, association of nephrectomy with survival in this cohort and may not transfer to other populations, and comparable performance of flexible and regression models is expected in small tabular datasets [52]. The comparison extended to accuracy and calibration: the two-variable Cox model had a lower integrated Brier score (0.164 vs. 0.189) and a calibration slope closer to 1 (0.79 vs. 0.54) than the network. The comparative conclusions were unchanged when the six patients who underwent nephrectomy after the start of first-line therapy were coded as not having undergone nephrectomy at baseline, although the contribution of nephrectomy was attenuated. This finding is informative in itself: it indicates that, in a cohort of this size, a parsimonious Cox model captures most of the available prognostic information, and that flexible models are unlikely to add value without substantially larger datasets. A conceptual feature of the network is the joint estimation of risk and survival time within a shared representation, whereas in Cox regression and random survival forests survival time must be derived post hoc from the survival curve; however, the accuracy of the predicted survival time was limited (MAE 23.9 months among patients who died) and was not compared with that of the benchmark models. Given the calibration slope below 1, the modest sample size and the lack of external validation, these results should be regarded as exploratory and hypothesis-generating.

This study has several strengths, including the integrated evaluation of established clinical risk models, routinely available inflammatory–nutritional biomarkers, and an exploratory neural-network approach within the same real-world mRCC cohort. The parallel use of conventional survival analyses and a neural-network model allowed prognostic associations and predictive contributions to be examined from two different perspectives, while nested repeated cross-validation with benchmarking against established and conventional models on identical splits provided an internal assessment of model performance. Nevertheless, several limitations should be acknowledged. The retrospective, single-center design and relatively modest sample size may limit generalizability and increase susceptibility to selection bias and residual confounding, particularly regarding the strong association observed with nephrectomy. Additional limitations include missing data requiring complete-case analysis in the conventional models, the absence of blinded central radiological review, median-derived cut-offs for PIV and HALP, potential model instability associated with backward stepwise selection, and the relatively small MSKCC poor-risk subgroup. Moreover, the effects of subsequent-line therapies could not be fully accounted for. The primary nephrectomy variable represented ever-nephrectomy and therefore incorporated post-baseline information for six patients, including five who underwent deferred nephrectomy after clinical benefit from systemic therapy. This definition is susceptible to immortal-time and responder-selection bias. Although sensitivity analyses based strictly on nephrectomy status at treatment initiation yielded directionally consistent associations and unchanged model comparisons, the effect estimates were attenuated, and the LOFO contribution of nephrectomy was approximately halved. Thus, the primary analysis may have overestimated the prognostic contribution of nephrectomy. Residual confounding by indication and patient selection preclude a causal interpretation of these findings. Given the small and clinically heterogeneous post-treatment subgroup, a landmark or time-dependent analysis was not performed.

In addition, the extended study period encompassed substantial changes in the therapeutic landscape, and only a small proportion of patients received contemporary ICI-based combinations, resulting in treatment-era heterogeneity. The inclusion of both clear-cell and non-clear-cell histologies further increased cohort heterogeneity. Finally, the neural network was developed with a small number of events relative to its number of parameters. Its risk predictions were overly extreme (calibration slope, 0.54), and its survival-probability estimates had greater prediction error than those of the Cox model with IMDC risk group and nephrectomy status (integrated Brier score, 0.189 vs. 0.164). Missing covariate values had to be imputed, survival-time accuracy could be assessed only among patients who died, and data-driven feature screening was unstable across folds. Although the network underwent nested repeated cross-validation, it was developed and evaluated within the same institutional cohort without external validation; its predictive performance should therefore be regarded as exploratory until confirmed in larger, independent, and contemporary datasets.

## 5. Conclusions

In this real-world mRCC cohort, established clinical risk stratification retained prognostic relevance, particularly for overall survival, while nephrectomy status emerged as the most consistently associated clinical factor across conventional and neural-network analyses. Inflammatory–nutritional markers, particularly PIV and HALP, were associated with overall survival in unadjusted analyses, but they did not show independent prognostic value after multivariable adjustment and did not improve discrimination in the neural-network analysis. The neural-network model achieved discrimination comparable to conventional Cox-based models, but its survival-probability estimates had greater prediction error than those of the two-variable Cox model and were poorly calibrated in slope, while its survival-time estimates had limited accuracy. Established risk models remained informative, whereas the association with nephrectomy should be interpreted cautiously because of potential immortal-time bias, responder selection, and residual confounding. Neither the inflammatory–nutritional indices nor the neural network provided clear incremental prognostic value beyond the parsimonious two-variable Cox model in this cohort. Recalibration and external validation in larger contemporary cohorts are required.

## Figures and Tables

**Table 1:** Baseline patient, disease, and treatment characteristics (*N* = 96).

Characteristic	Value	Characteristic	Value
**Demographic and clinical characteristics**		**Disease characteristics**	
Age, years, mean ± SD	62.1 ± 10.9	Histological subtype ^d^	
Male sex	72 (75.0)	Clear-cell RCC	68 (70.8)
KPS, median (IQR)	100 (80–100)	Non-clear-cell RCC	19 (19.8)
KPS 100	61 (63.5)	Missing	9 (9.4)
KPS 90	6 (6.2)	Timing of metastatic disease	
KPS 80	22 (22.9)	Synchronous	55 (57.3)
KPS 70	7 (7.3)	Metachronous	41 (42.7)
Smoking history	41 (42.7)	Number of metastatic sites ^e^	
Alcohol use	8 (8.3)	1	47 (49.0)
≥1 recorded comorbidity	70 (72.9)	≥2	48 (50.0)
**Prognostic risk**		Metastatic sites ^e^	
IMDC risk group		Lung	63 (65.6)
Favorable	17 (17.7)	Bone	48 (50.0)
Intermediate	60 (62.5)	Lymph node	13 (13.5)
Poor	19 (19.8)	Brain	10 (10.4)
MSKCC risk group ^a^		Adrenal	9 (9.4)
Favorable	16 (16.7)	Liver	8 (8.3)
Intermediate	68 (70.8)	Peritoneum	3 (3.1)
Poor	11 (11.5)	Other	11 (11.5)
Individual IMDC/MSKCC risk factors ^b^		Nephrectomy	
Diagnosis to systemic therapy < 1 year	59 (61.5)	None	27 (28.1)
KPS < 80	7 (7.3)	Before metastatic presentation	41 (42.7)
Hemoglobin < LLN	53 (55.2)	Upfront cytoreductive	22 (22.9)
Corrected calcium > ULN	6 (6.2)	Deferred cytoreductive, after systemic therapy	5 (5.2)
Neutrophils > ULN	12 (12.5)	Palliative, after systemic therapy	1 (1.0)
Platelets > ULN	16 (16.7)	**Treatment**	
LDH > 1.5 × ULN	9 (9.5) ^c^	First-line systemic therapy	
**Baseline laboratory and inflammatory–nutritional measures, median (IQR)**		Sunitinib	43 (44.8)
Neutrophils, ×10^9^/L	4.46 (3.29–5.71)	Pazopanib	23 (24.0)
Lymphocytes, ×10^9^/L	1.82 (1.38–2.53)	Cabozantinib	20 (20.8)
Monocytes, ×10^9^/L	0.54 (0.40–0.74)	Nivolumab	1 (1.0)
Platelets, ×10^9^/L	236.4 (193.5–313.0)	Nivolumab plus cabozantinib	9 (9.4)
Hemoglobin, g/dL	12.4 (11.3–13.7)	Second-line systemic therapy ^f^	
Albumin, g/dL	4.2 (3.7–4.4)	None	41 (42.7)
Corrected calcium, mg/dL	9.2 (8.9–9.5)	VEGFR-TKI monotherapy	27 (28.1)
LDH, U/L	214 (184–261) ^c^	ICI monotherapy	24 (25.0)
PIV	326.0 (169.4–528.6)	mTOR inhibitor	2 (2.1)
HALP score	34.7 (22.9–47.2)	ICI-based combination	2 (2.1)

Values are *n* (%) unless otherwise indicated; percentages use *N* = 96 unless otherwise specified. For binary variables without missing data, only one category is shown. ^a^ Not assessable in 1 patient (1.0%). ^b^ Thresholds correspond to the institutional reference limits applied in the IMDC and MSKCC classifications; corrected calcium values and sex-specific hemoglobin limits were used. ^c^ Available for 95 patients; the percentage uses *n* = 95 as the denominator. ^d^ Detailed non-clear-cell subtypes and sarcomatoid features were not consistently available in the retrospective records. ^e^ Assessed at metastatic diagnosis; patients could have more than one site. Vena cava involvement was not classified as a metastatic site. Number of sites unavailable for 1 patient (1.0%). ^f^ Therapies initiated during follow-up rather than baseline exposure. Abbreviations: HALP, hemoglobin–albumin–lymphocyte–platelet; ICI, immune checkpoint inhibitor; IMDC, International Metastatic Renal Cell Carcinoma Database Consortium; IQR, interquartile range; KPS, Karnofsky Performance Status; LDH, lactate dehydrogenase; LLN, lower limit of normal; MSKCC, Memorial Sloan Kettering Cancer Center; mTOR, mammalian target of rapamycin; PIV, pan-immune-inflammation value; RCC, renal cell carcinoma; SD, standard deviation; TKI, tyrosine kinase inhibitor; ULN, upper limit of normal; VEGFR, vascular endothelial growth factor receptor.

**Table 2:** Multivariable Cox proportional hazards regression analyses for progression-free survival.

Model	Variable/Comparison	HR	95% CI	*p* Value
Model initially including IMDC	No nephrectomy vs. nephrectomy	2.25	1.32–3.81	**0.003**
MSKCC-based model	No nephrectomy vs. nephrectomy	2.25	1.32–3.84	**0.003**
Score-free model	No nephrectomy vs. nephrectomy	2.24	1.32–3.81	**0.003**

Abbreviations: CI, confidence interval; HR, hazard ratio; IMDC, International Metastatic Renal Cell Carcinoma Database Consortium; MSKCC, Memorial Sloan Kettering Cancer Center; PFS, progression-free survival. Note: Cox proportional hazards regression models were constructed using backward stepwise selection based on the likelihood ratio criterion. The poor-risk category was the reference group for the MSKCC classification, and patients who underwent nephrectomy constituted the reference category for nephrectomy status. Age was entered as a continuous covariate in the initial models. In the score-free model, KPS was also included initially. Statistically significant *p* values are shown in bold. The model-level sample sizes, numbers of PFS events, and Harrell’s C-indices were as follows: IMDC-containing model, *n* = 96, 77 events, C-index = 0.570; MSKCC-containing model, *n* = 95, 76 events, C-index = 0.570; and score-free model, *n* = 96, 77 events, C-index = 0.570.

**Table 3:** Multivariable Cox proportional hazards regression analyses for overall survival.

Model	Variable/Comparison	HR	95% CI	*p* Value
IMDC-based model	IMDC risk group, overall	—	—	0.024
	Favorable vs. poor	0.27	0.09–0.79	0.016
	Intermediate vs. poor	0.45	0.23–0.87	0.017
	Age, per 1-year increase	1.03	1.00–1.05	0.060
	No nephrectomy vs. nephrectomy	4.48	2.33–8.60	<0.001
MSKCC-based model	MSKCC risk group, overall	—	—	**0.032**
	Favorable vs. poor	0.26	0.09–0.78	**0.016**
	Intermediate vs. poor	0.40	0.18–0.89	**0.024**
	Age, per 1-year increase	1.02	1.00–1.05	0.103
	PIV > 325.97 vs. ≤325.97	1.66	0.91–3.04	0.101
	No nephrectomy vs. nephrectomy	4.66	2.37–9.16	**<0.001**
Score-free model	KPS ≤ 80 vs. >80	2.31	1.25–4.28	**0.008**
	PIV > 325.97 vs. ≤325.97	1.64	0.93–2.92	0.090
	No nephrectomy vs. nephrectomy	3.79	1.94–7.42	**<0.001**

Abbreviations: CI, confidence interval; HR, hazard ratio; IMDC, International Metastatic Renal Cell Carcinoma Database Consortium; KPS, Karnofsky Performance Status; MSKCC, Memorial Sloan Kettering Cancer Center; OS, overall survival; PIV, pan-immune-inflammation value. Note: Cox proportional hazards regression models were constructed using backward stepwise selection based on the likelihood ratio criterion. The table presents all variables retained in the final models. The poor-risk category was used as the reference group for IMDC and MSKCC classifications. Patients who underwent nephrectomy, patients with PIV ≤ 325.97, and patients with KPS > 80 constituted the respective reference categories. HRs and 95% CIs are reported to two decimal places and *p* values to three decimal places; values below 0.001 are reported as *p* < 0.001. Statistically significant *p* values are shown in bold. The model-level sample sizes, numbers of deaths, and Harrell’s C-indices were as follows: IMDC-based model, *n* = 95, 57 deaths, C-index = 0.710; MSKCC-based model, *n* = 94, 56 deaths, C-index = 0.715; and score-free model, *n* = 95, 57 deaths, C-index = 0.714.

**Table 4:** Discrimination of the neural network and comparator models for overall survival on identical nested cross-validation splits (stratified 5-fold cross-validation repeated 10 times; 50 outer folds).

Model	Fold-Wise C-Index, Mean (SD)	Pooled Out-of-Fold C-Index (95% CI)	ΔC, Network–Model (95% CI)
Kaplan–Meier null model	0.500 (0.000)	0.500 (0.500–0.500)	0.185 (0.108 to 0.264)
IMDC risk group (Cox)	0.639 (0.070)	0.629 (0.542–0.711)	0.055 (−0.011 to 0.123)
MSKCC risk group (Cox)	0.602 (0.058)	0.594 (0.501–0.681)	0.090 (0.003 to 0.175)
IMDC risk group + nephrectomy (Cox)	0.713 (0.075)	0.692 (0.608–0.773)	−0.007 (−0.060 to 0.046)
Cox, network-selected predictors	0.665 (0.076)	0.673 (0.591–0.752)	0.012 (−0.023 to 0.048)
Cox, all 13 candidates (ridge)	0.666 (0.072)	0.668 (0.584–0.748)	0.017 (−0.017 to 0.052)
Elastic-net (LASSO-type) Cox, all candidates	0.685 (0.062)	0.685 (0.609–0.761)	−0.001 (−0.027 to 0.025)
Random survival forest, all candidates	0.694 (0.071)	0.695 (0.616–0.769)	−0.010 (−0.036 to 0.018)
Neural network (primary, nested screening)	0.679 (0.084)	0.685 (0.608–0.764)	—
Neural network, all 13 candidates	0.687 (0.072)	0.690 (0.613–0.768)	−0.005 (−0.017 to 0.008)

Abbreviations: C, Harrell’s concordance index; CI, confidence interval; IMDC, International Metastatic Renal Cell Carcinoma Database Consortium; MSKCC, Memorial Sloan Kettering Cancer Center; OOF, out-of-fold. Note: All models were fitted on the same outer training folds and evaluated on the same held-out folds. The predictors of the Cox model with network-selected predictors differ between folds, because screening was repeated within each training fold. Pooled OOF C was computed from fold-standardized risk scores averaged across repeats; CIs of the C-index and of ΔC are from 1000 patient-level bootstrap resamples. Fold-level results are given in Table S4.

**Table 5:** Overall accuracy and calibration of the neural network, the Cox model with IMDC risk group and nephrectomy status, the random survival forest and the Kaplan–Meier null model (nested cross-validation; 50 outer folds).

Metric	Neural Network	Cox, IMDC Risk Group + Nephrectomy	Random Survival Forest	Kaplan–Meier Null Model
IPCW Brier score, 6 months	0.099	0.097	0.100	0.103
IPCW Brier score, 12 months	0.172	0.156	0.167	0.197
IPCW Brier score, 24 months	0.189	0.161	0.181	0.222
IPCW Brier score, 36 months	0.213	0.181	0.208	0.251
Integrated Brier score, 3–60 months	0.189	0.164	0.183	0.221
Calibration-in-the-large, 12/24/36 months	−0.009/−0.012/−0.028	0.003/0.001/−0.009	0.005/0.008/0.003	—
Calibration slope (ideal value 1)	0.54 (SD 0.22)	0.79 (SD 0.06)	1.04 (SD 0.11) ^a^	—

Abbreviations: IPCW, inverse probability of censoring weighting. Note: Lower Brier scores indicate more accurate predicted survival probabilities. Calibration-in-the-large is the mean predicted minus the observed Kaplan–Meier survival; calibration slope is the mean of the slopes from Cox regression of observed survival on the out-of-fold linear predictor, estimated separately in each of the 10 repeats (SD across repeats in parentheses). The Kaplan–Meier null model assigns the same survival probability to every patient. ^a^ For the random survival forest, which has no linear predictor, the calibration slope was estimated from the complementary log-log of its predicted 24-month survival.

## Data Availability

The data presented in this study are available on request from the corresponding author due to institutional and patient privacy restrictions. The complete analysis code for the neural-network and benchmark analyses is openly available on Zenodo at https://doi.org/10.5281/zenodo.23515898.

## Supplementary Materials

The following supporting information can be downloaded at: https://www.mdpi.com/article/10.3390/jcm15218262/s1, Supplementary Methods S1: Neural-network architecture, training, validation and performance measures; Table S1: Progression-free survival according to demographic, clinical, disease-related, treatment-related, and inflammatory–nutritional characteristics; Table S2: Overall survival according to demographic, clinical, disease-related, treatment-related, and inflammatory–nutritional characteristics; Table S3: Neural-network feature screening and hyperparameter selection, including selection frequency, full-cohort permutation importance, leave-one-feature-out contribution, and hyperparameter combinations selected across the 50 outer folds; Table S4: Fold-level Harrell’s C-index of all models for overall survival across the 50 outer folds; Table S5: Sensitivity analysis of neural-network and comparator-model discrimination for overall survival after recoding six patients who underwent nephrectomy after initiation of first-line therapy as not having undergone nephrectomy at baseline; Table S6: Sensitivity analyses using nephrectomy status at initiation of first-line systemic therapy; Figure S1: Kaplan–Meier curves for progression-free survival according to IMDC risk group; Figure S2: Kaplan–Meier curves for progression-free survival according to MSKCC risk group; Figure S3: Kaplan–Meier curves for progression-free survival according to nephrectomy status; Figure S4: Kaplan–Meier curves for overall survival according to IMDC risk group; Figure S5: Kaplan–Meier curves for overall survival according to MSKCC risk group; Figure S6: Kaplan–Meier curves for overall survival according to nephrectomy status; Figure S7: Selection frequency of the 13 candidate predictors across the 50 outer training folds; Figure S8: Time-dependent IPCW Brier scores of the neural network, the Cox model with IMDC risk group and nephrectomy status, the random survival forest, and the Kaplan–Meier null model; TRIPOD + AI checklist.

## References

[1] European Society for Medical Oncology (2026). ESMO Living Guideline: Renal Cell Carcinoma. Management of Advanced and Metastatic Renal Cell Carcinoma.

[2] Powles T., Burotto M., Escudier B., Apolo A., Bourlon M., Shah A., Suárez C., Porta C., Barrios C., Richardet M. (2024). Nivolumab plus cabozantinib versus sunitinib for first-line treatment of advanced renal cell carcinoma: Extended follow-up from the phase III randomized CheckMate 9ER trial. ESMO Open.

[3] Motzer R.J., Porta C., Eto M., Powles T., Grünwald V., E Hutson T., Alekseev B., Rha S.Y., Merchan J., Goh J.C. (2024). Lenvatinib plus pembrolizumab versus sunitinib in first-line treatment of advanced renal cell carcinoma: Final prespecified overall survival analysis of CLEAR, a phase III study. J. Clin. Oncol..

[4] Heng D.Y.C., Xie W., Regan M.M., Warren M.A., Golshayan A.R., Sahi C., Eigl B.J., Ruether J.D., Cheng T., North S. (2009). Prognostic factors for overall survival in patients with metastatic renal cell carcinoma treated with vascular endothelial growth factor-targeted agents: Results from a large, multicenter study. J. Clin. Oncol..

[5] Heng D.Y.C., Xie W., Regan M.M., Harshman L.C., Bjarnason G.A., Vaishampayan U.N., Mackenzie M., Wood L., Donskov F., Tan M.-H. (2013). External validation and comparison with other models of the International Metastatic Renal-Cell Carcinoma Database Consortium prognostic model: A population-based study. Lancet Oncol..

[6] Ernst M.S., Navani V., Wells J.C., Donskov F., Basappa N., Labaki C., Pal S.K., Meza L., Wood L.A., Ernst D.S. (2023). Outcomes for International Metastatic Renal Cell Carcinoma Database Consortium prognostic groups in contemporary first-line combination therapies for metastatic renal cell carcinoma. Eur. Urol..

[7] Kikuta M., Naito S., Osawa T., Numakura K., Narisawa T., Takai Y., Yagi M., Sekine Y., Tokairin O., Shinohara N. (2025). Real-world short-term outcomes and treatment regimen comparisons in patients with metastatic renal cell carcinoma treated with first-line immune combinations. BMC Cancer.

[8] Motzer R.J., Bacik J., Murphy B.A., Russo P., Mazumdar M. (2002). Interferon-alfa as a comparative treatment for clinical trials of new therapies against advanced renal cell carcinoma. J. Clin. Oncol..

[9] Mantia C.M., Jegede O.A., McDermott D.F., Heng D.Y.C., Xie W., Choueiri T.K., Atkins M.B., Regan M.M. (2026). Long-term performance of prognostic models for advanced renal cell carcinoma in the era of improved survival with immune checkpoint inhibitors. JCO Oncol. Pract..

[10] Tomčová Z., Obertová J., Chovanec M., Syčová-Milá Z., Štefániková K., Šlachtová E., Žák M., Savka A., Hrnčár M., Rejleková K. (2025). Prognostic value of hemoglobin, albumin, lymphocyte, platelet (HALP) score in patients with metastatic renal cell carcinoma treated with nivolumab. Biomedicines.

[11] Yekedüz E., Tural D., Ertürk I., Karakaya S., Erol C., Ercelep Ö., Arslan Ç., Sever Ö.N., Kılıçkap S., Öztaş N.Ş. (2022). The relationship between pan-immune-inflammation value and survival outcomes in patients with metastatic renal cell carcinoma treated with nivolumab in the second line and beyond: A Turkish Oncology Group Kidney Cancer Consortium study. J. Cancer Res. Clin. Oncol..

[12] Fucà G., Guarini V., Antoniotti C., Morano F., Moretto R., Corallo S., Marmorino F., Lonardi S., Rimassa L., Sartore-Bianchi A. (2020). The Pan-Immune-Inflammation Value is a new prognostic biomarker in metastatic colorectal cancer: Results from a pooled-analysis of the Valentino and TRIBE first-line trials. Br. J. Cancer.

[13] Guven D.C., Sahin T.K., Erul E., Kilickap S., Gambichler T., Aksoy S. (2022). The association between the pan-immune-inflammation value and cancer prognosis: A systematic review and meta-analysis. Cancers.

[14] Chen X.L., Xue L., Wang W., Chen H.N., Zhang W.H., Liu K., Chen X.-Z., Yang K., Zhang B., Chen Z.-X. (2015). Prognostic significance of the combination of preoperative hemoglobin, albumin, lymphocyte and platelet in patients with gastric carcinoma: A retrospective cohort study. Oncotarget.

[15] Ma W., Liu W., Dong Y., Zhang J., Hao L., Xia T., Wang X., Han C. (2024). Predicting the prognosis of patients with renal cell carcinoma based on the systemic immune inflammation index and prognostic nutritional index. Sci. Rep..

[16] Katzman J.L., Shaham U., Cloninger A., Bates J., Jiang T., Kluger Y. (2018). DeepSurv: Personalized treatment recommender system using a Cox proportional hazards deep neural network. BMC Med. Res. Methodol..

[17] Barkan E., Porta C., Rabinovici-Cohen S., Tibollo V., Quaglini S., Rizzo M. (2023). Artificial intelligence-based prediction of overall survival in metastatic renal cell carcinoma. Front. Oncol..

[18] Chandramohan D., Garapati H.N., Nangia U., Simhadri P.K., Lapsiwala B., Jena N.K., Singh P. (2024). Diagnostic accuracy of deep learning in detection and prognostication of renal cell carcinoma: A systematic review and meta-analysis. Front. Med..

[19] Cox D.R. (1972). Regression models and life-tables. J. R. Stat. Soc. Ser. B Stat. Methodol..

[20] Huber P.J., Kotz S., Johnson N.L. (1992). Robust estimation of a location parameter. Breakthroughs in Statistics: Methodology and Distribution.

[21] Breiman L. (2001). Random forests. Mach. Learn..

[22] Van der Ploeg T., Austin P.C., Steyerberg E.W. (2014). Modern modelling techniques are data hungry: A simulation study for predicting dichotomous endpoints. BMC Med. Res. Methodol..

[23] Riley R.D., Ensor J., Snell K.I.E., E Harrell F., Martin G.P., Reitsma J.B., Moons K.G.M., Collins G., van Smeden M. (2020). Calculating the sample size required for developing a clinical prediction model. BMJ.

[24] Varma S., Simon R. (2006). Bias in error estimation when using cross-validation for model selection. BMC Bioinform..

[25] Tibshirani R. (1997). The lasso method for variable selection in the Cox model. Stat. Med..

[26] Ishwaran H., Kogalur U.B., Blackstone E.H., Lauer M.S. (2008). Random survival forests. Ann. Appl. Stat..

[27] Harrell F.E., Califf R.M., Pryor D.B., Lee K.L., Rosati R.A. (1982). Evaluating the yield of medical tests. JAMA.

[28] Efron B., Tibshirani R.J. (1994). An Introduction to the Bootstrap.

[29] Graf E., Schmoor C., Sauerbrei W., Schumacher M. (1999). Assessment and comparison of prognostic classification schemes for survival data. Stat. Med..

[30] Breslow N.E. (1972). Discussion of Professor Cox’s paper. J. R. Stat. Soc. Ser. B Stat. Methodol..

[31] Van Calster B., McLernon D.J., van Smeden M., Wynants L., Steyerberg E.W. (2019). Calibration: The Achilles heel of predictive analytics. BMC Med..

[32] Paszke A., Gross S., Massa F., Lerer A., Bradbury J., Chanan G., Killeen T., Lin Z., Gimelshein N., Antiga L. (2019). PyTorch: An imperative style, high-performance deep learning library. Adv. Neural Inf. Process Syst..

[33] Davidson-Pilon C. (2019). Lifelines: Survival analysis in Python. J. Open Source Softw..

[34] Pölsterl S. (2020). Scikit-survival: A library for time-to-event analysis built on top of scikit-learn. J. Mach. Learn Res..

[35] Collins G.S., Moons K.G.M., Dhiman P., Riley R.D., Beam A.L., Van Calster B., Ghassemi M., Liu X., Reitsma J.B., van Smeden M. (2024). TRIPOD+AI statement: Updated guidance for reporting clinical prediction models that use regression or machine learning methods. BMJ.

[36] Panian J., Lin X., Simantov R., Derweesh I., Choueiri T.K., McKay R.R. (2020). The Impact of Age and Gender on Outcomes of Patients With Advanced Renal Cell Carcinoma Treated With Targeted Therapy. Clin. Genitourin. Cancer.

[37] Rini B.I., Plimack E.R., Stus V., Gafanov R., Waddell T., Nosov D., Pouliot F., Alekseev B., Soulières D., Melichar B. (2025). Pembrolizumab plus axitinib versus sunitinib for advanced clear cell renal cell carcinoma: 5-year survival and biomarker analyses of the phase 3 KEYNOTE-426 trial. Nat. Med..

[38] Motzer R.J., Tannir N.M., McDermott D.F., Arén Frontera O., Melichar B., Choueiri T.K., Plimack E.R., Barthélémy P., Porta C., George S. (2018). Nivolumab plus Ipilimumab versus Sunitinib in Advanced Renal-Cell Carcinoma. N. Engl. J. Med..

[39] Shah N.J., Sura S.D., Shinde R., Shi J., Singhal P.K., Robert N.J., Vogelzang N.J., Perini R.F., Motzer R.J. (2023). Real-world Treatment Patterns and Clinical Outcomes for Metastatic Renal Cell Carcinoma in the Current Treatment Era. Eur. Urol. Open Sci..

[40] Motzer R.J., Hutson T.E., Cella D., Reeves J., Hawkins R., Guo J., Nathan P., Staehler M., De Souza P., Merchan J.R. (2013). Pazopanib versus sunitinib in metastatic renal-cell carcinoma. N. Engl. J. Med..

[41] Choueiri T.K., Halabi S., Sanford B.L., Hahn O., Michaelson M.D., Walsh M.K., Feldman D.R., Olencki T., Picus J., Small E.J. (2017). Cabozantinib versus sunitinib as initial targeted therapy for patients with metastatic renal cell carcinoma of poor or intermediate risk: The Alliance A031203 CABOSUN trial. J. Clin. Oncol..

[42] Bolek H., Kuzu O.F., Sertesen Camoz E., Sim S., Sekmek S., Karakas H., Isık S., Guliyev M., Akkus A.F., Tural D. (2025). Sunitinib in metastatic renal cell carcinoma: Clinical outcomes across risk groups in a Turkish Oncology Group Kidney Cancer Consortium. Cancer Commun..

[43] Sagie S., Sarfaty M., Levartovsky M., Gantz Sorotsky H., Berger R., Percik R., Gadot M. (2022). RCC Real-World Data: Prognostic Factors and Risk Stratification in the Immunotherapy Era. Cancers.

[44] Takemura K., Lemelin A., Ernst M.S., Wells J.C., Saliby R.M., El Zarif T., Labaki C., Basappa N.S., Szabados B., Powles T. (2024). Outcomes of Patients with Brain Metastases from Renal Cell Carcinoma Receiving First-line Therapies: Results from the International Metastatic Renal Cell Carcinoma Database Consortium. Eur. Urol..

[45] Heng D.Y.C., Wells J.C., Rini B.I., Beuselinck B., Lee J.L., Knox J.J., Bjarnason G.A., Pal S.K., Kollmannsberger C.K., Yuasa T. (2014). Cytoreductive nephrectomy in patients with synchronous metastases from renal cell carcinoma: Results from the International Metastatic Renal Cell Carcinoma Database Consortium. Eur. Urol..

[46] Bakouny Z., El Zarif T., Dudani S., Connor Wells J., Gan C.L., Donskov F., Shapiro J., Davis I.D., Parnis F., Ravi P. (2023). Upfront cytoreductive nephrectomy for metastatic renal cell carcinoma treated with immune checkpoint inhibitors or targeted therapy: An observational study from the International Metastatic Renal Cell Carcinoma Database Consortium. Eur. Urol..

[47] Méjean A., Ravaud A., Thezenas S., Colas S., Beauval J.-B., Bensalah K., Geoffrois L., Thiery-Vuillemin A., Cormier L., Lang H. (2018). Sunitinib Alone or after Nephrectomy in Metastatic Renal-Cell Carcinoma. N. Engl. J. Med..

[48] Bex A., Mulders P., Jewett M., Wagstaff J., Van Thienen J.V., Blank C.U., Van Velthoven R., Del Pilar Laguna M., Wood L., Van Melick H.H.E. (2019). Comparison of Immediate vs Deferred Cytoreductive Nephrectomy in Patients With Synchronous Metastatic Renal Cell Carcinoma Receiving Sunitinib: The SURTIME Randomized Clinical Trial. JAMA Oncol..

[49] Makrakis D., Msaouel P., Karam J.A., Esagian S.M. (2025). Cytoreductive Nephrectomy in Metastatic Renal Cell Carcinoma Treated with Immune Checkpoint Inhibitors: A Systematic Review and Meta-analysis of Individual Patient Data. Eur. Urol. Focus..

[50] Günaltılı M., Bölek H., Kaplan Tüzün E., Usta S., Efil S.C., Akkus A.F., Işık S., Kapar C., Sekmek S., Güzel H.G. (2026). Pretreatment HALP Score and Survival Outcomes in Patients with Metastatic Renal Cell Carcinoma Receiving First-Line Tyrosine Kinase Inhibitors: A Turkish Oncology Group Kidney Cancer Consortium (TKCC) Study. Cancers.

[51] Güzel H.G., Aydın A.A., Hacıçavuşoğlu A., Yazdan Balçık O., Beypınar İ., Öztürk B., Yıldız M. (2026). Evaluation of the predictive and prognostic performance of the PILE score in metastatic renal cell carcinoma. Front. Med..

[52] Christodoulou E., Ma J., Collins G.S., Steyerberg E.W., Verbakel J.Y., Van Calster B. (2019). A systematic review shows no performance benefit of machine learning over logistic regression for clinical prediction models. J. Clin. Epidemiol..

