# Supplementary material for: Clinical Risk Models, Inflammatory–Nutritional Indices and a Neural-Network Survival Model in Metastatic Renal Cell Carcinoma: A Single-Center Retrospective Study
Source: J Clin Med. 2026 Oct 23;15(21):8262. doi: 10.3390/jcm15218262 (PMC13764327; doi:10.3390/jcm15218262)
Supplement: Supplementary file 1 [file jcm-15-08262-s001.zip › jcm-4563873-supplementary.pdf]

SUPPLEMENTARY MATERIALS

Supplementary Methods S1. Neural-network architecture, training, validation and performance measures

S1.1 Candidate predictors and preprocessing

Thirteen candidate predictors were considered: age at diagnosis (years, continuous), male sex (0/1), any comorbidity (0/1), nephrectomy status (0/1), the IMDC factor count (0–6, ordinal), the MSKCC factor count (0–5, ordinal), the natural logarithm of PIV, HALP (continuous), mean platelet volume (MPV, continuous), clear-cell histology (0/1), and bone, lung and brain metastasis (each 0/1). KPS and the diagnosis-to-treatment interval were not entered separately because they are components of the IMDC and MSKCC scores; first-line treatment was not entered because it is a treatment decision rather than a baseline patient characteristic and was highly imbalanced (TKI monotherapy, n = 86; ICI-based therapy, n = 10).

Histology was missing in 9 patients and bone metastasis in 1 patient. In one patient, LDH was missing. Because the network requires complete inputs, the MSKCC factor count of this patient was derived from the remaining MSKCC components (KPS <80%, diagnosis-to-treatment interval <1 year, hemoglobin <13 g/dL in men and <12 g/dL in women, calcium >10.2 mg/dL), i.e., LDH was assumed to be normal, giving two factors (intermediate risk); in the conventional analyses, this patient was treated as missing for MSKCC. All other preprocessing was fitted on the training data of each fold only and then applied to the corresponding held-out data: continuous and ordinal variables were imputed with the training-fold median and scaled with a robust scaler (median and interquartile range of the training fold), and binary variables were imputed with the training-fold mode.

S1.2 Architecture and loss functions

The network was a fully connected feed-forward network (multilayer perceptron) with a shared trunk of two hidden layers: input → linear (32 units) → ReLU → dropout (0.3) → linear (16 units) → ReLU → dropout (0.2). Two linear output heads were attached to the trunk: a risk head (one unit, no bias) producing a log-risk score, and a time head (one unit, with bias) predicting log(1 + survival time in months). The network has 913 trainable parameters with 10 inputs and 1009 with all 13 inputs.

The risk head was trained with the negative Cox partial log-likelihood (Breslow handling of ties), divided by the number of events. The time head was trained with a censoring-aware Huber loss on log(1 + months) with threshold δ: for patients who died, the full Huber loss of the residual was used; for censored patients, only predictions shorter than the censoring time were penalized (the residual was truncated at zero). The total loss was the Cox loss + $\lambda \times$ Huber loss + an L1 penalty (sum of the absolute values of all weight matrices multiplied by the L1 coefficient).

S1.3 Optimization

All networks were trained full-batch with AdamW and gradient-norm clipping at 5.0 on CPU with fixed random seeds. During screening and hyperparameter tuning, networks were trained for a fixed number of epochs (80 and 100, respectively). The final network of each outer fold was trained on 85% of the outer training set for up to 300 epochs with cosine annealing of the learning rate, using the remaining 15% (stratified by event status) for early stopping (patience 30 epochs on the combined validation loss; the weights with the lowest validation loss were retained). The time head was then linearly recalibrated on the same 15% internal split, using the patients who died (slope restricted to 0.7–1.3 and intercept to ±0.5; applied only when at least 5 deaths were available), as in the original analysis.

S1.4 Feature screening

Within each outer training set, a stratified 3-fold inner cross-validation was performed. In each inner fold, a risk-only network (Cox loss) and a time-only network (censoring-aware Huber loss) were trained (learning rate 0.01, weight decay 0.01, $\delta = 1.0$, 80 epochs), and the permutation importance of each candidate was computed on the inner validation data as the mean decrease in Harrell's C-index (risk head) and the mean increase in Huber loss (time head) over three random permutations. A candidate was retained if its mean importance over the three inner folds was positive for either head; if fewer than five candidates met this rule, the five with the highest importance were retained. Screening therefore used only the outer training data. On average, 10.0 of the 13 candidates were retained per outer fold; selection frequencies are given in Table S3 and Figure S7.

S1.5 Hyperparameter tuning

Hyperparameters were tuned by grid search within a second stratified 3-fold inner cross-validation of the outer training set, using the screened predictors. The grid comprised the Huber threshold δ (0.1, 0.5), the L1 coefficient (0.005, 0.01), the weight decay (0.001, 0.005) and the time-loss weight λ (2, 5), with a learning rate of 0.005 (16 combinations). Among the combinations whose mean inner C-index lay within 0.015 of the best (the tolerance was widened in steps of 0.005 up to 0.04 until at least five combinations qualified), the combination with the lowest mean inner Huber loss was selected. The selected combinations are given in Table S3.

S1.6 Outer validation and benchmark models

Performance was estimated with stratified (by event status) 5-fold cross-validation repeated 10 times (50 outer folds; random seed 2026). Screening, tuning and fitting were repeated within each outer training set, and all models were evaluated on the same held-out folds. The benchmark models were: (i) a Kaplan–Meier null model; (ii) Cox models with the IMDC risk group, the MSKCC risk group, or the IMDC risk group plus nephrectomy status (risk groups entered as ordinal variables: favorable 0, intermediate 1, poor 2); (iii) a Cox model with the predictors selected by the network screening in the same fold; (iv) a ridge-penalized Cox model with all 13 candidates (penalty 0.05); (v) an elastic-net (LASSO-type) Cox model with all candidates (L1 ratio 0.9; penalty selected by inner 3-fold cross-validation on the C-index); and (vi) a random survival forest with all candidates (300 trees, minimum 8 patients per leaf, square-root feature sampling). The Cox models in (ii) and (iii) used a small ridge penalty (0.01) for numerical stability. A network trained on all 13 candidates without screening, with the hyperparameters selected in the same fold, served as a sensitivity analysis. In a second sensitivity analysis, the six patients who underwent nephrectomy after the start of first-line therapy—five deferred nephrectomies after clinical benefit and one palliative nephrectomy—were coded as not having undergone nephrectomy at baseline, and the complete nested analysis, including screening, tuning, all benchmark models, and the LOFO analysis, was repeated using the identical 50 outer folds (Table S5).

S1.7 Performance measures

Survival probabilities of the network and the Cox models were obtained from the out-of-fold linear predictor and the Breslow baseline cumulative hazard estimated in the corresponding training data. Discrimination was quantified with Harrell's C-index in each outer fold (fold-wise mean and SD) and as a pooled out-of-fold estimate: in each repeat, the held-out risk scores were standardized with the mean and SD of the risk scores of the corresponding training fold, and the standardized scores were averaged across the 10 repeats. 95% CIs of the pooled C-index and of the paired difference between the network and each comparator (ΔC) were obtained from 1000 patient-level bootstrap resamples.

Overall accuracy of predicted survival probabilities (averaged across repeats) was assessed with inverse probability of censoring weighted (IPCW) Brier scores at 6, 12, 24 and 36 months and with the integrated Brier score over 3–60 months (monthly grid, trapezoidal rule), with the censoring distribution estimated by the reverse Kaplan–Meier method; the Kaplan–Meier null model, which assigns the same survival probability to every patient, served as the reference. Calibration was assessed with calibration-in-the-large (mean predicted

minus observed Kaplan–Meier survival) at 12, 24 and 36 months, with the calibration slope (coefficient of the out-of-fold linear predictor in a Cox model, estimated separately in each repeat; mean 0.54, SD 0.22 over the 10 repeats; 0.52, SD 0.14, without screening), and with calibration plots by tertiles of predicted survival (Figure 5). The mean and median absolute errors of the predicted survival time (back-transformed, averaged across repeats) were computed in the 57 patients who died, because the true survival time of censored patients is unknown; these metrics therefore exclude censored patients and are biased toward shorter survival. Brier scores, the integrated Brier score, calibration-in-the-large, and the calibration slope were also calculated for the Cox model with IMDC risk group and nephrectomy status and for the random survival forest; because the forest has no linear predictor, its calibration slope was estimated from the complementary log-log transformation of its predicted 24-month survival.

S1.8 Leave-one-feature-out analysis

For interpretation only, screening and tuning were repeated on the full cohort, which retained 10 predictors (age, male sex, nephrectomy status, IMDC and MSKCC factor counts, log PIV, HALP, MPV, clear-cell histology and brain metastasis; selected hyperparameters δ 0.5, learning rate 0.005, L1 coefficient 0.01, weight decay 0.005, λ 5). Each predictor was removed in turn, and the network was refitted (100 epochs without early stopping, with the full-cohort hyperparameters) and evaluated in 5 × 5-fold cross-validation (25 folds) paired with the full model; the decrease in C-index is reported as mean ± SD (Figure 4; Table S3). This full-cohort model was not used to estimate performance.

S1.9 Software and code

Analyses were performed in Python 3.9.13 with PyTorch 2.8.0, NumPy 2.0.2, pandas 2.3.3, scikit-learn 1.5.2, lifelines 0.30.0, scikit-survival 0.23.1 and matplotlib 3.9.4. The complete analysis code (version 16.2) is available on Zenodo at https://doi.org/10.5281/zenodo.23515898. Patient-level data are not shared because of patient-privacy restrictions.

Table S1. Progression-free survival according to demographic, clinical, disease-related, treatment-related, and inflammatory–nutritional characteristics

**Characteristics**	**Category**	**n**	**PFS events, n**	**Median PFS, months**	**95% CI**	***p* value**
**Demographic and clinical characteristics**						
Sex	Female	24	18	7.3	0.00–14.86	0.289
	Male	72	59	19.1	10.65–27.55	
Age	<65 years	54	43	17.7	10.96–24.44	0.949
	≥65 years	42	34	19.1	1.14–37.06	
Karnofsky Performance Status	>80	67	52	23.0	14.43–31.57	**0.025**
	≤80	29	25	7.9	4.21–11.59	
Smoking status	No	55	45	17.7	8.32–27.08	0.698
	Yes	41	32	18.7	5.79–31.61	
Alcohol consumption	No	88	70	17.7	8.36–27.04	0.753
	Yes	8	7	18.7	7.89–29.51	
**Comorbidities**						
Any comorbidity	No	15	12	21.6	1.44–41.76	0.840
	Yes	69	56	18.7	8.72–28.68	
**Disease-related characteristics**						
Histological subtype	Clear cell	68	55	15.2	6.45–23.95	0.540
	Other	19	15	13.5	0.00–32.46	
Bone metastasis	No	47	37	18.7	1.90–35.50	0.373
	Yes	48	40	15.2	4.12–26.28	
Lung metastasis	No	33	25	17.7	5.74–29.66	0.871
	Yes	63	52	17.8	9.38–26.22	
Brain metastasis	No	86	68	19.1	8.98–29.22	0.146
	Yes	10	9	10.0	3.65–16.35	
**Prognostic risk classifications**						
IMDC risk group	Favorable	17	12	26.5	19.20–NE	**0.027**
	Intermediate	60	48	17.7	6.05–29.35	
	Poor	19	17	7.9	3.63–12.17	
MSKCC risk group	Favorable	16	12	26.5	18.50–34.50	0.284
	Intermediate	68	54	13.8	2.94–24.66	
	Poor	11	10	7.0	3.66–10.35	
**Treatment-related characteristics**						
First-line treatment category	TKI	86	72	15.2	8.47–21.93	0.357
	IO or combination therapy	10	5	21.6	3.57–39.63	
TKI agent	Sunitinib	43	40	17.8	10.99–24.61	0.909
	Pazopanib	23	20	13.5	0.00–31.95	
	Cabozantinib	20	12	10.8	4.88–16.72	
Diagnosis-to-treatment interval	≥1 year	37	28	26.5	24.99–28.01	0.208
	<1 year	59	49	11.1	8.00–14.21	

Characteristics	Category	n	PFS events, n	Median PFS, months	95% CI	*p* value
Nephrectomy	No	27	23	10.0	7.12–12.88	**0.002**
	Yes	69	54	26.4	17.92–34.88	
**Inflammatory and nutritional biomarkers**						
PIV	≤325.97	47	38	26.4	16.46–36.34	0.330
	>325.97	49	39	10.8	7.74–13.86	
HALP	>34.68	49	38	19.1	6.91–31.29	0.167
	≤34.68	47	39	13.8	6.25–21.35	
MPV category	Normal	51	37	18.7	1.36–36.04	0.388
	Low	33	32	13.8	6.94–20.67	
	High	12	8	19.1	0.00–39.74	

Abbreviations: CI, confidence interval; HALP, hemoglobin-albumin-lymphocyte-platelet score; IMDC, International Metastatic Renal Cell Carcinoma Database Consortium; IO, immunotherapy; MPV, mean platelet volume; MSKCC, Memorial Sloan Kettering Cancer Center; PFS, progression-free survival; PIV, pan-immune-inflammation value; TKI, tyrosine kinase inhibitor. NE, not estimable.

Note: p values were obtained using the log-rank test. Statistically significant values are shown in bold. For IMDC, unadjusted pairwise comparisons were favorable vs intermediate, p = 0.361; favorable vs poor, p = 0.013; and intermediate vs poor, p = 0.031. For MSKCC, the corresponding comparisons were favorable vs intermediate, p = 0.308; favorable vs poor, p = 0.113; and intermediate vs poor, p = 0.306.

The number of patients included in individual analyses may vary because of missing data.

Table S2. Overall survival according to demographic, clinical, disease-related, treatment-related, and inflammatory–nutritional characteristics

Characteristics	Category	n	Deaths, n	Median OS, months	95% CI	*p* value
**Demographic and clinical characteristics**						
Sex	Female	24	14	57.3	11.52–103.08	0.232
	Male	72	43	46.3	10.82–81.78	
Age	<65 years	54	31	42.6	26.95–58.25	0.954
	≥65 years	42	26	60.6	27.14–94.07	
Karnofsky Performance Status	>80	67	35	70.5	50.71–90.29	**<0.001**
	≤80	29	22	12.0	0.00–33.99	
Smoking status	No	55	35	45.2	17.52–72.88	0.128
	Yes	41	22	72.7	28.39–117.01	
Alcohol consumption	No	88	52	46.3	20.47–72.13	0.580
	Yes	8	5	76.9	28.61–125.19	
**Comorbidities**						
Any comorbidity	No	15	8	46.3	20.62–71.98	0.839
	Yes	69	42	59.5	22.69–96.31	
**Disease-related characteristics**						
Histological subtype	Clear cell	68	39	59.5	28.50–90.50	0.208
	Other	19	12	32.0	17.38–46.62	
Bone metastasis	No	47	24	64.9	31.92–97.89	**0.042**

Characteristics	Category	n	Deaths, n	Median OS, months	95% CI	*p* value
	Yes	48	33	39.9	26.20–53.60	
Lung metastasis	No	33	21	33.4	22.08–44.72	0.161
	Yes	63	36	70.5	49.27–91.74	
Brain metastasis	No	86	48	59.5	32.69–86.31	**0.034**
	Yes	10	9	11.4	7.68–15.12	
**Prognostic risk classifications**						
IMDC risk group	Favorable	17	6	95.4	79.98 – 110.82	**0.001**
	Intermediate	60	36	45.2	20.36 – 70.04	
	Poor	19	15	9.1	6.68 –11.52	
MSKCC risk group	Favorable	16	6	95.4	79.97–110.83	**0.018**
	Intermediate	68	42	42.6	18.03–67.17	
	Poor	11	8	9.6	0.00–26.63	
**Treatment-related characteristics**						
First-line treatment category	TKI	86	53	57.3	32.90–81.70	0.888
	IO or combination therapy	10	4	28.0	—	
TKI agent	Sunitinib	43	29	59.5	27.12–91.88	0.527
	Pazopanib	23	16	57.3	17.60–97.00	
	Cabozantinib	20	8	Not reached	—	
Diagnosis-to-treatment interval	≥1 year	37	18	76.8	53.18–100.42	**0.043**
	<1 year	59	39	33.2	14.14–52.26	
Nephrectomy	No	27	22	11.9	6.98–16.82	**<0.001**
	Yes	69	35	76.8	62.63–90.97	
**Inflammatory and nutritional biomarkers**						
PIV	≤325.97	47	25	64.9	45.83–83.97	**0.032**
	>325.97	49	32	33.4	21.49–45.31	
HALP	>34.68	49	26	81.2	37.01–125.39	**0.040**
	≤34.68	47	31	36.0	21.40–50.60	
MPV category	Normal	51	29	70.5	22.04–118.96	0.604
	Low	33	23	42.6	25.17–60.03	
	High	12	5	59.5	0.00–136.39	

Abbreviations: CI, confidence interval; HALP, hemoglobin-albumin-lymphocyte-platelet score; IMDC, International Metastatic Renal Cell Carcinoma Database Consortium; IO, immunotherapy; MPV, mean platelet volume; MSKCC, Memorial Sloan Kettering Cancer Center; OS, overall survival; PIV, pan-immune-inflammation value; TKI, tyrosine kinase inhibitor.

Note: p values were obtained using the log-rank test. Statistically significant values are shown in bold. For IMDC, unadjusted pairwise comparisons were favorable vs intermediate, $p = 0.061$; favorable vs poor, $p = 0.003$; and intermediate vs poor, $p = 0.006$. For MSKCC, the corresponding comparisons were favorable vs intermediate, $p = 0.043$; favorable vs poor, $p = 0.037$; and intermediate vs poor, $p = 0.073$.

The number of patients included in individual analyses may vary because of missing data.

Table S3. Neural-network feature screening and hyperparameter selection. (A) Selection frequency of the 13 candidate predictors across the 50 outer training folds, permutation importance in the full-cohort screening, and leave-one-feature-out (LOFO) contribution in the full-cohort model.

Predictor	Selected in outer folds, %	Full-cohort importance, risk head (ΔC)	Full-cohort importance, time head (Δ Huber loss)	Retained in full-cohort model	LOFO ΔC, mean ± SD
Nephrectomy	100	0.0599	0.0849	Yes	0.044 ± 0.052
log PIV	94	−0.0097	0.0089	Yes	−0.002 ± 0.033
MSKCC factors (0-5)	92	0.0044	0.0031	Yes	0.002 ± 0.023
Clear-cell histology	90	0.0032	−0.0012	Yes	0.007 ± 0.028
Age (years)	88	−0.0100	0.0003	Yes	−0.006 ± 0.032
HALP	82	0.0128	0.0028	Yes	−0.011 ± 0.024
Brain metastasis	76	0.0111	−0.0004	Yes	−0.002 ± 0.025
Bone metastasis	74	−0.0021	−0.0090	No	—
IMDC factors (0-6)	72	0.0195	−0.0126	Yes	−0.004 ± 0.027
Male sex	68	0.0059	−0.0048	Yes	−0.004 ± 0.038
MPV	66	0.0007	−0.0136	Yes	−0.006 ± 0.030
Any comorbidity	52	−0.0061	−0.0151	No	—
Lung metastasis	50	−0.0136	−0.0215	No	—

Predictors are ordered by selection frequency. A predictor was retained if its mean permutation importance was positive for the risk head (decrease in C-index) or the time head (increase in censoring-aware Huber loss). LOFO ΔC is the mean ± SD decrease in validation C-index over 25 paired folds (5 × 5-fold cross-validation) when the predictor is removed from the full-cohort model; — , not retained in the full-cohort model. The full-cohort model is used for interpretation only. PIV, pan-immune-inflammation value; HALP, hemoglobin–albumin–lymphocyte–platelet score; MPV, mean platelet volume.

Table S3 (continued). (B) Hyperparameter combinations selected in the 50 outer folds.

Huber δ	L1 coefficient	Weight decay	Time-loss weight λ	Learning rate	Outer folds, n (of 50)
0.1	0.01	0.005	5	0.005	25
0.1	0.01	0.001	5	0.005	15
0.1	0.01	0.001	2	0.005	3
0.1	0.005	0.005	5	0.005	2
0.1	0.005	0.005	2	0.005	2
0.1	0.01	0.005	2	0.005	1
0.1	0.005	0.001	5	0.005	1
0.5	0.01	0.001	5	0.005	1

Selection rule: lowest inner-validation Huber loss among combinations whose inner C-index was within 0.015 (widened up to 0.04) of the best. Hyperparameters selected in the full-cohort model (LOFO only): δ 0.5, L1 coefficient 0.01, weight decay 0.005, λ 5, learning rate 0.005.

Table S4. Fold-level Harrell's C-index of all models for overall survival across the 50 outer folds (stratified 5-fold cross-validation repeated 10 times; identical splits for all models).

Repeat	Fold	NN inputs, n	IMDC	MSKCC	IMDC + Neph	Cox, NN-sel.	Cox, all	EN-Cox	RSF	NN	NN, no scr.
1	1	10	0.637	0.537	0.725	0.617	0.633	0.650	0.633	0.617	0.633
1	2	11	0.621	0.598	0.661	0.679	0.670	0.705	0.696	0.723	0.714
1	3	10	0.571	0.533	0.679	0.633	0.617	0.662	0.558	0.658	0.650
1	4	10	0.720	0.648	0.678	0.669	0.669	0.703	0.720	0.720	0.695
1	5	10	0.659	0.667	0.736	0.696	0.696	0.717	0.739	0.746	0.746
2	1	12	0.643	0.673	0.665	0.699	0.692	0.688	0.677	0.737	0.714
2	2	8	0.784	0.685	0.824	0.703	0.640	0.721	0.721	0.721	0.703
2	3	11	0.603	0.528	0.762	0.667	0.659	0.694	0.786	0.730	0.714
2	4	6	0.491	0.491	0.616	0.593	0.620	0.630	0.565	0.556	0.602
2	5	9	0.686	0.659	0.690	0.581	0.651	0.667	0.659	0.558	0.690
3	1	11	0.512	0.580	0.640	0.456	0.480	0.512	0.584	0.424	0.488
3	2	11	0.705	0.563	0.760	0.677	0.717	0.709	0.732	0.685	0.732
3	3	11	0.540	0.567	0.623	0.619	0.595	0.603	0.619	0.587	0.603
3	4	11	0.692	0.654	0.825	0.800	0.800	0.825	0.833	0.767	0.825
3	5	8	0.702	0.605	0.685	0.653	0.685	0.694	0.710	0.774	0.742
4	1	8	0.609	0.645	0.645	0.633	0.672	0.629	0.641	0.656	0.641
4	2	9	0.739	0.682	0.826	0.659	0.705	0.674	0.841	0.758	0.780
4	3	12	0.596	0.562	0.715	0.677	0.662	0.708	0.631	0.638	0.615
4	4	10	0.681	0.638	0.724	0.664	0.690	0.759	0.741	0.759	0.776
4	5	11	0.611	0.558	0.745	0.558	0.538	0.577	0.615	0.558	0.548
5	1	7	0.640	0.544	0.765	0.632	0.581	0.640	0.669	0.632	0.632
5	2	12	0.643	0.605	0.677	0.639	0.662	0.707	0.707	0.684	0.677
5	3	12	0.566	0.566	0.624	0.619	0.628	0.668	0.637	0.699	0.726

Repeat	Fold	NN inputs, n	IMDC	MSKCC	IMDC + Neph	Cox, NN-sel.	Cox, all	EN-Cox	RSF	NN	NN, no scr.
5	4	12	0.590	0.671	0.686	0.733	0.705	0.705	0.695	0.724	0.724
5	5	12	0.698	0.631	0.802	0.794	0.786	0.778	0.746	0.786	0.810
6	1	9	0.651	0.643	0.869	0.849	0.817	0.710	0.833	0.841	0.794
6	2	9	0.661	0.673	0.730	0.726	0.677	0.774	0.774	0.766	0.694
6	3	9	0.614	0.575	0.667	0.623	0.719	0.662	0.632	0.632	0.667
6	4	5	0.696	0.513	0.634	0.638	0.670	0.670	0.661	0.629	0.723
6	5	7	0.588	0.596	0.673	0.677	0.685	0.692	0.685	0.654	0.638
7	1	11	0.578	0.578	0.810	0.816	0.789	0.711	0.769	0.728	0.776
7	2	8	0.743	0.622	0.757	0.670	0.670	0.722	0.704	0.626	0.678
7	3	12	0.650	0.581	0.711	0.585	0.545	0.650	0.667	0.593	0.593
7	4	12	0.643	0.638	0.647	0.598	0.634	0.652	0.679	0.607	0.634
7	5	12	0.575	0.575	0.675	0.658	0.675	0.711	0.719	0.658	0.675
8	1	13	0.544	0.492	0.659	0.611	0.595	0.635	0.627	0.643	0.643
8	2	10	0.635	0.611	0.627	0.698	0.619	0.671	0.690	0.683	0.730
8	3	8	0.661	0.610	0.760	0.685	0.654	0.772	0.803	0.787	0.811
8	4	11	0.694	0.641	0.714	0.650	0.621	0.650	0.680	0.680	0.680
8	5	11	0.690	0.649	0.819	0.710	0.734	0.718	0.710	0.718	0.702
9	1	11	0.775	0.697	0.906	0.779	0.779	0.742	0.803	0.779	0.713
9	2	11	0.590	0.567	0.705	0.629	0.638	0.686	0.705	0.619	0.638
9	3	7	0.484	0.516	0.587	0.595	0.603	0.556	0.540	0.579	0.579
9	4	10	0.676	0.588	0.698	0.802	0.794	0.729	0.733	0.779	0.733
9	5	10	0.695	0.650	0.752	0.659	0.691	0.732	0.732	0.748	0.715
10	1	13	0.645	0.605	0.688	0.558	0.587	0.707	0.688	0.746	0.746
10	2	12	0.557	0.478	0.635	0.652	0.635	0.609	0.635	0.583	0.609

Repeat	Fold	NN inputs, n	IMDC	MSKCC	IMDC + Neph	Cox, NN-sel.	Cox, all	EN-Cox	RSF	NN	NN, no scr.
10	3	11	0.694	0.694	0.806	0.621	0.641	0.709	0.689	0.631	0.583
10	4	6	0.561	0.548	0.557	0.579	0.588	0.557	0.605	0.526	0.623
10	5	10	0.734	0.663	0.805	0.809	0.809	0.823	0.759	0.794	0.773
Mean (SD)		10.0	0.639 (0.070)	0.602 (0.058)	0.713 (0.075)	0.665 (0.076)	0.666 (0.072)	0.685 (0.062)	0.694 (0.071)	0.679 (0.084)	0.687 (0.072)
NN better, % of folds			70	88	30	50	48	44	38	—	40

The Kaplan–Meier null model has a C-index of 0.500 in every fold and is not shown. Mean (SD) is over the 50 folds; "NN better" is the percentage of folds in which the primary network had a higher C-index than the comparator. Fold-wise C-indices are based on about 19 patients and 11 deaths per fold and are therefore highly variable; pooled out-of-fold estimates with bootstrap CIs are given in Table 4. EN-Cox, elastic-net (LASSO-type) Cox model; IMDC, IMDC risk group (Cox); IMDC + Neph, IMDC risk group plus nephrectomy status (Cox); MSKCC, MSKCC risk group (Cox); NN, neural network (primary, nested screening); NN, no scr., neural network with all 13 candidates; NN inputs, number of predictors retained by screening; NN-sel., predictors selected by the network screening in the same fold; RSF, random survival forest.

Table S5. Sensitivity analysis of neural-network and comparator-model discrimination for overall survival after recoding six patients who underwent nephrectomy after initiation of first-line therapy as not having undergone nephrectomy at baseline

Model	Pooled OOF C-index (95% CI), primary analysis	Pooled OOF C-index (95% CI), sensitivity analysis	ΔC, network − model (95% CI), sensitivity analysis
Kaplan–Meier null model	0.500 (0.500–0.500)	0.500 (0.500–0.500)	0.177 (0.101 to 0.258)
IMDC risk group (Cox)	0.629 (0.542–0.711)	0.629 (0.542–0.711)	0.047 (−0.013 to 0.110)
MSKCC risk group (Cox)	0.594 (0.501–0.681)	0.594 (0.501–0.681)	0.082 (−0.008 to 0.168)
IMDC risk group + nephrectomy (Cox)	0.692 (0.608–0.773)	0.672 (0.585–0.760)	0.005 (−0.056 to 0.062)
Cox, network-selected predictors	0.673 (0.591–0.752)	0.651 (0.564–0.733)	0.025 (−0.013 to 0.062)
Cox, all 13 candidates (ridge)	0.668 (0.584–0.748)	0.650 (0.564–0.734)	0.027 (−0.010 to 0.065)
Elastic-net (LASSO-type) Cox, all candidates	0.685 (0.609–0.761)	0.665 (0.588–0.748)	0.011 (−0.010 to 0.036)
Random survival forest, all candidates	0.695 (0.616–0.769)	0.672 (0.585–0.752)	0.005 (−0.021 to 0.032)
Neural network (primary, nested screening)	0.685 (0.608–0.764)	0.677 (0.601–0.758)	—
Neural network, all 13 candidates	0.690 (0.613–0.768)	0.678 (0.599–0.759)	−0.001 (−0.013 to 0.011)

In the sensitivity analysis, five patients with deferred nephrectomy after clinical benefit and one patient with palliative nephrectomy were coded as “no nephrectomy at baseline” (nephrectomy, 63 vs 69 patients in the primary analysis); screening, tuning, all benchmark models and the LOFO analysis were repeated. Nephrectomy status was retained in 100% of outer folds and remained the predictor with the largest LOFO contribution (ΔC, 0.024 ± 0.048; primary analysis, 0.044 ± 0.052). Integrated Brier score / calibration slope: neural network 0.192 / 0.60; Cox model with IMDC risk group and nephrectomy status 0.177 / 0.81; random survival forest 0.191 / 0.92 (primary analysis: 0.189 / 0.54, 0.164 / 0.79 and 0.183 / 1.04). The IMDC and MSKCC risk-group models do not include nephrectomy and are therefore unchanged. Abbreviations: C, Harrell’s concordance index; CI, confidence interval; LOFO, leave-one-feature-out; OOF, out-of-fold.

Table S6. Sensitivity analyses using nephrectomy status at initiation of first-line systemic therapy

Outcome	Analysis	Comparison	Effect estimate	95% CI	*P* value
**PFS**	Kaplan–Meier	No baseline nephrectomy (n=33) vs baseline nephrectomy (n=63)	Median: 10.9 vs 23.0 months	7.81–13.99 vs 14.02–31.98	**0.015**
**PFS**	IMDC-based Cox model	No baseline nephrectomy vs baseline nephrectomy	HR 1.70	1.03–2.80	**0.038**
**PFS**	MSKCC-based Cox model	No baseline nephrectomy vs baseline nephrectomy	HR 1.82	1.11–2.98	**0.018**
**PFS**	Score-free Cox model	No baseline nephrectomy vs baseline nephrectomy	HR 1.66	1.00–2.75	0.050
**OS**	Kaplan–Meier	No baseline nephrectomy (n=33) vs baseline nephrectomy (n=63)	Median: 17.7 vs 72.7 months	4.39–31.01 vs 54.10–91.30	**<0.001**
**OS**	IMDC-based Cox model	No baseline nephrectomy vs baseline nephrectomy	HR 2.78	1.52–5.09	**<0.001**
**OS**	MSKCC-based Cox model	No baseline nephrectomy vs baseline nephrectomy	HR 2.63	1.42–4.90	**0.002**
**OS**	Score-free Cox model	No baseline nephrectomy vs baseline nephrectomy	HR 2.26	1.23–4.17	**0.009**

Note: In the primary analyses, nephrectomy was defined as ever-nephrectomy. For this sensitivity analysis, nephrectomy status was redefined according to status at initiation of first-line systemic therapy. The six patients who underwent nephrectomy after treatment initiation were therefore classified as having no nephrectomy at baseline; this classification does not imply that they were never nephrectomized. Cox estimates are from the corresponding final backward likelihood-ratio models.

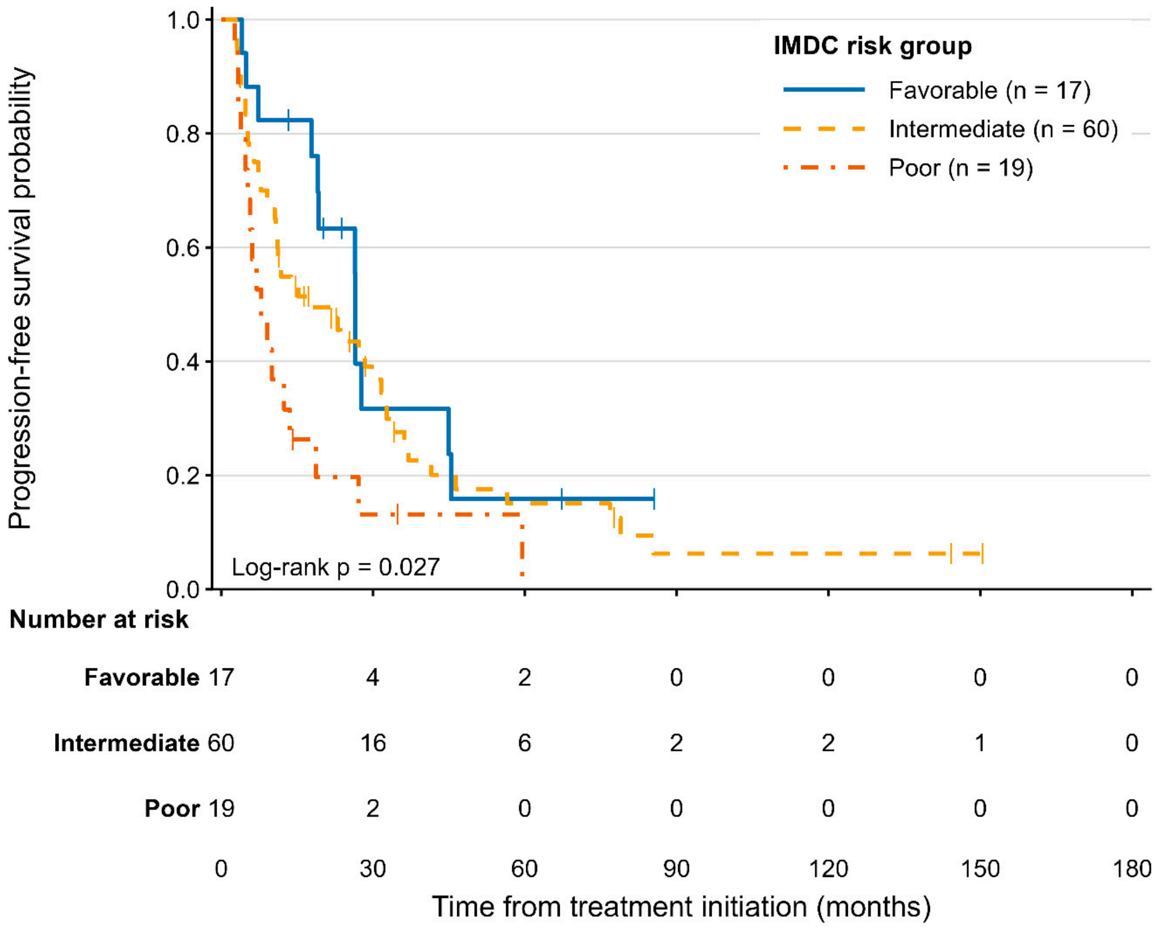

Figure S1. Kaplan–Meier curves for progression-free survival according to IMDC risk group. Tick marks indicate censored observations; numbers at risk are shown beneath the x-axis.

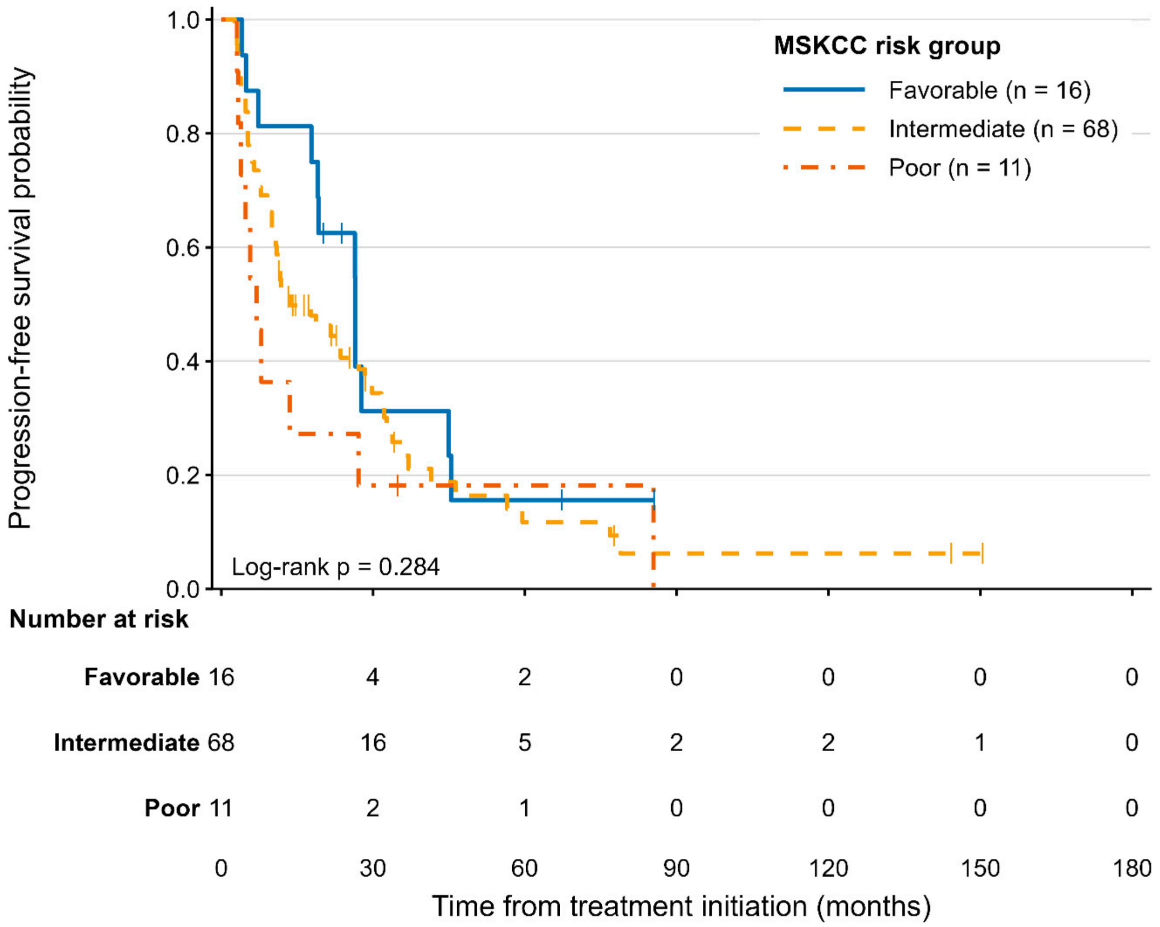

Figure S2. Kaplan–Meier curves for progression-free survival according to MSKCC risk group. Tick marks indicate censored observations; numbers at risk are shown beneath the x-axis.

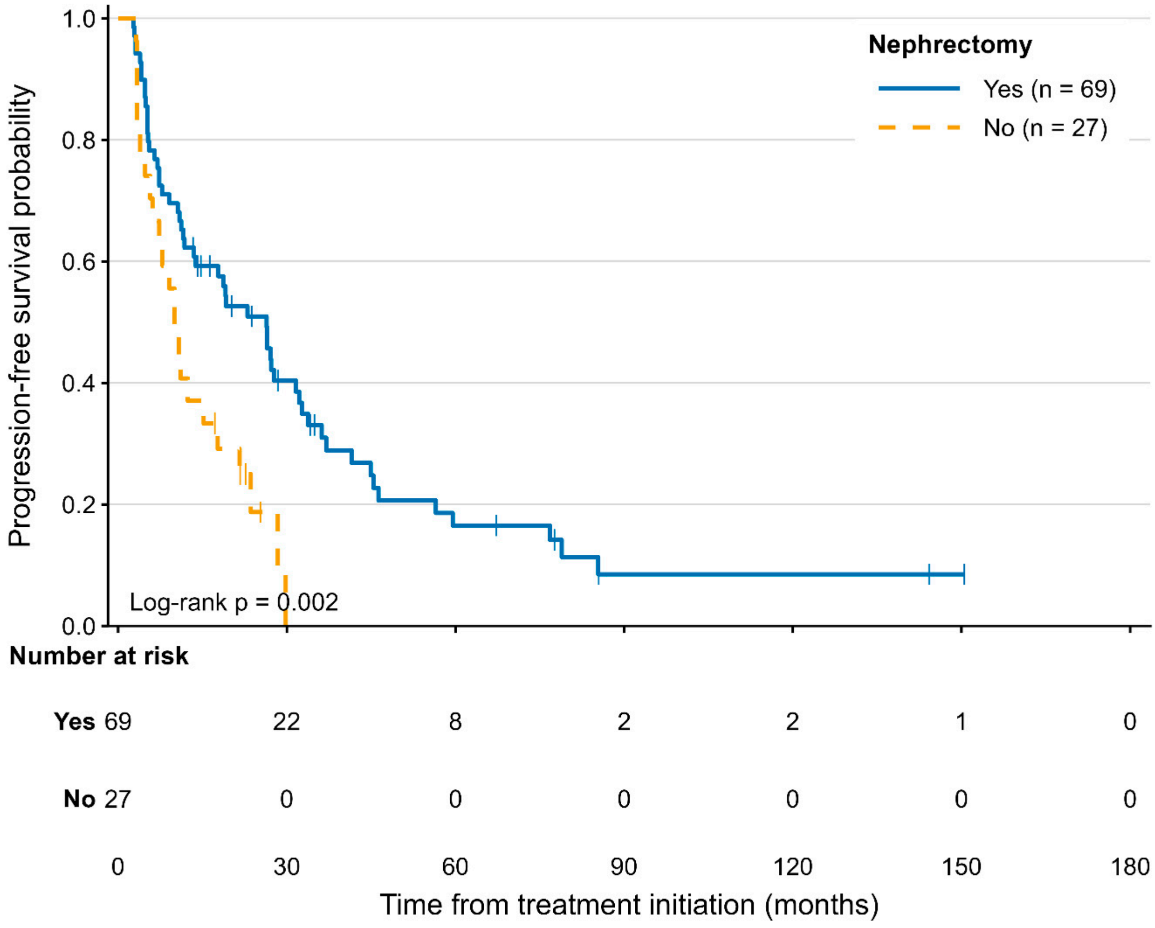

Figure S3. Kaplan–Meier curves for progression-free survival according to nephrectomy status. Tick marks indicate censored observations; numbers at risk are shown beneath the x-axis.

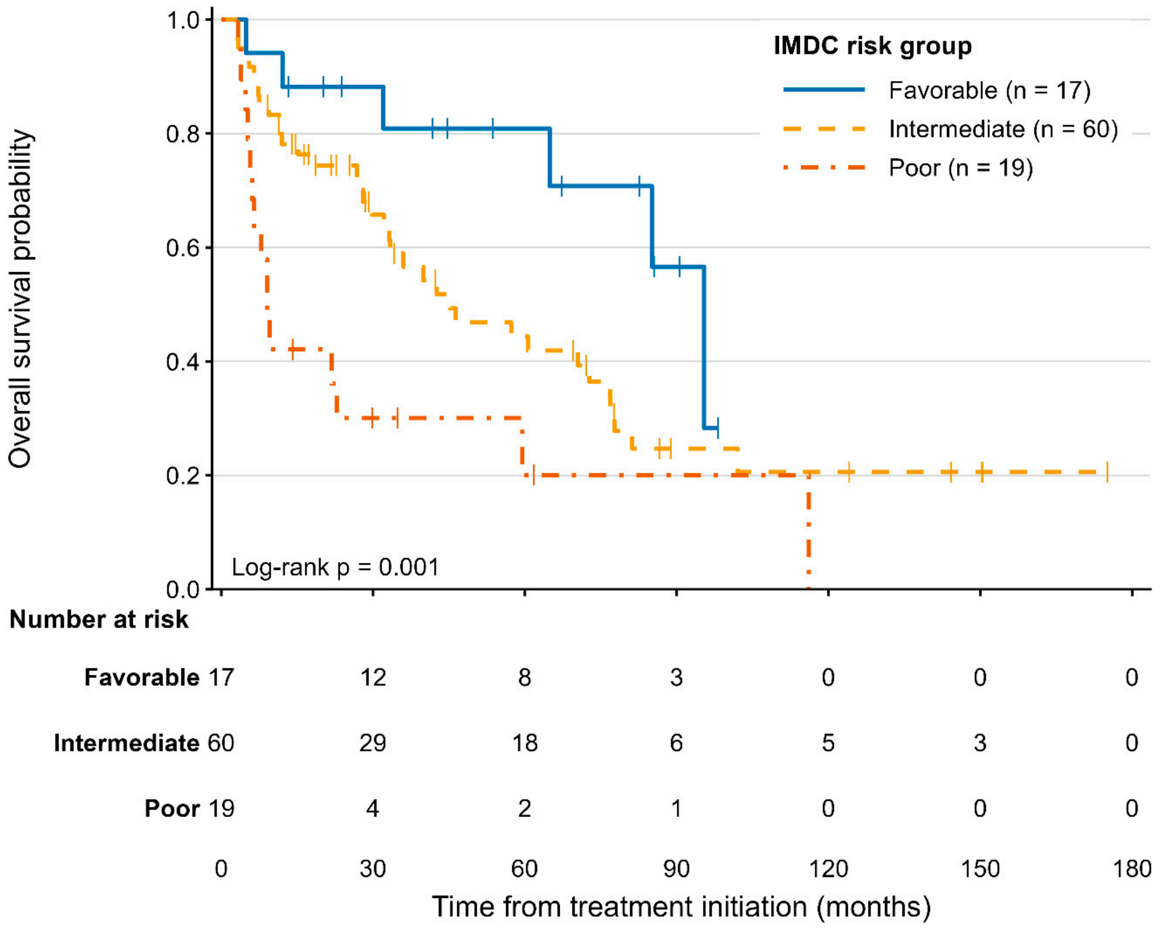

Figure S4. Kaplan–Meier curves for overall survival according to IMDC risk group. Tick marks indicate censored observations; numbers at risk are shown beneath the x-axis.

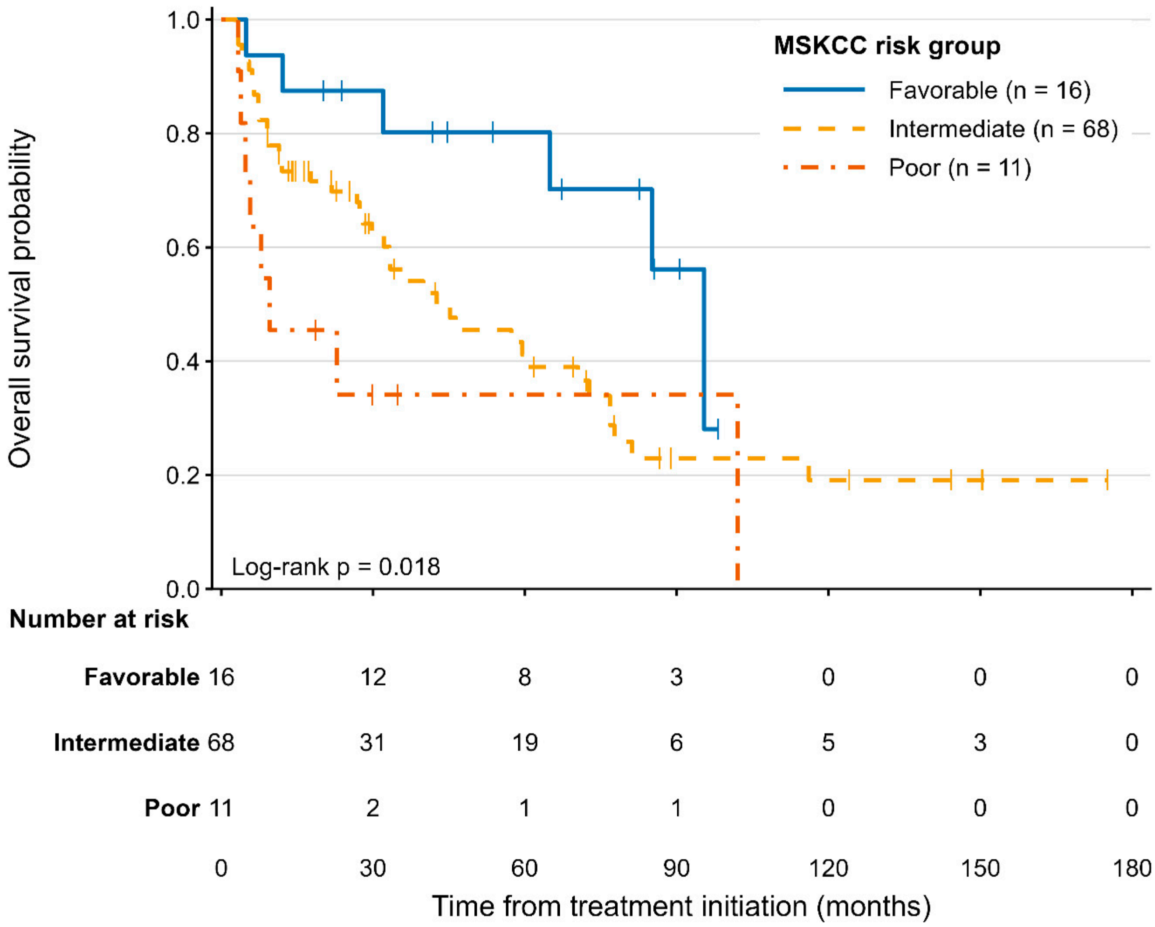

Figure S5. Kaplan–Meier curves for overall survival according to MSKCC risk group. Tick marks indicate censored observations; numbers at risk are shown beneath the x-axis.

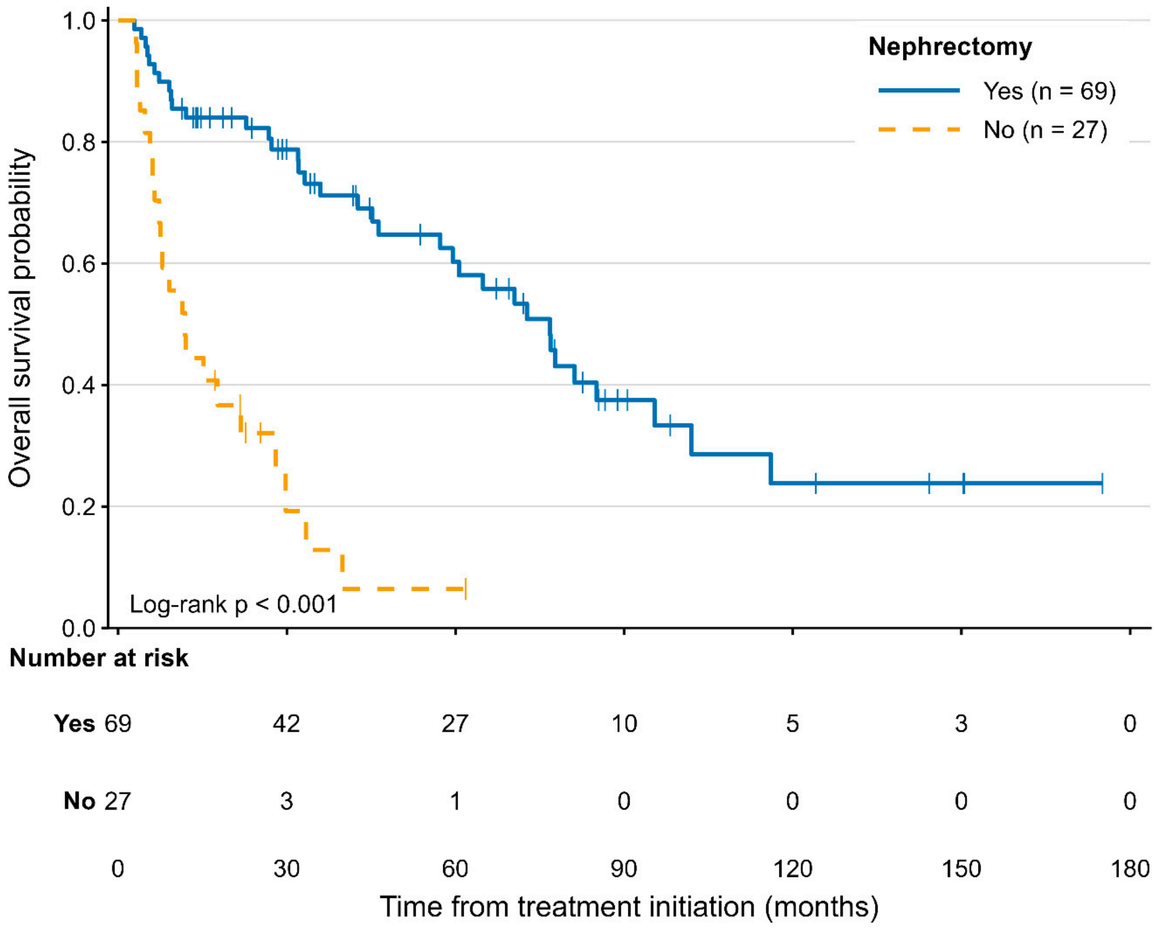

Figure S6. Kaplan–Meier curves for overall survival according to nephrectomy status. Tick marks indicate censored observations; numbers at risk are shown beneath the x-axis.

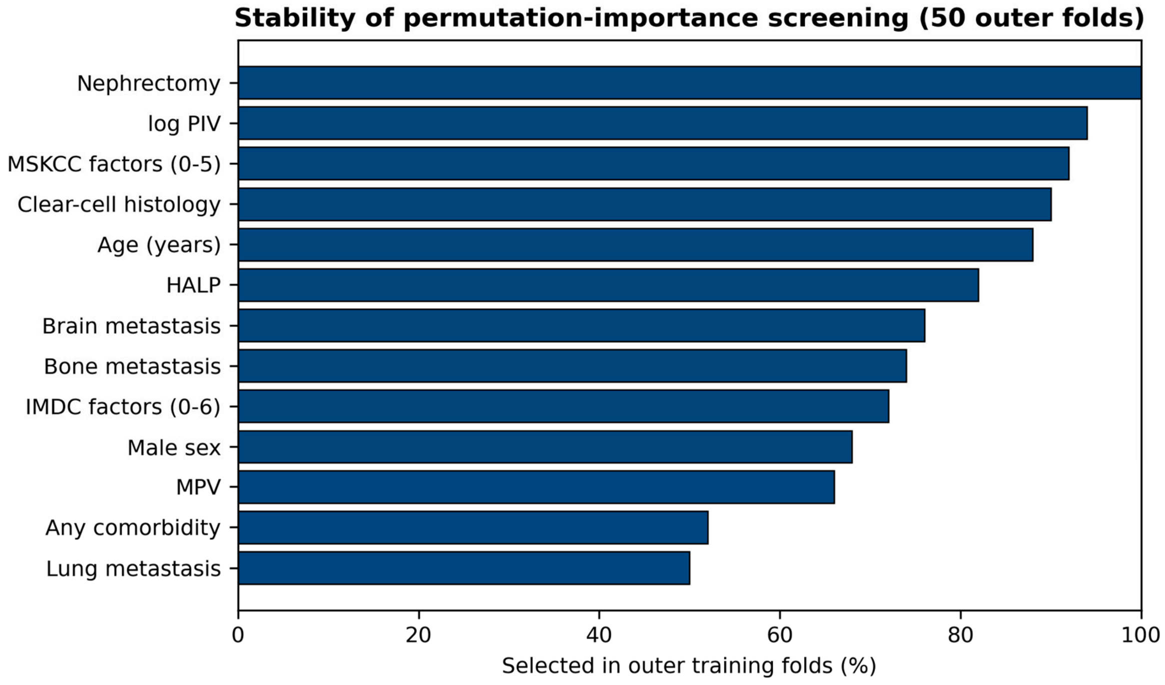

Figure S7. Selection frequency of the 13 candidate predictors in the permutation-importance screening performed within each of the 50 outer training folds. Only nephrectomy status was selected in every fold.

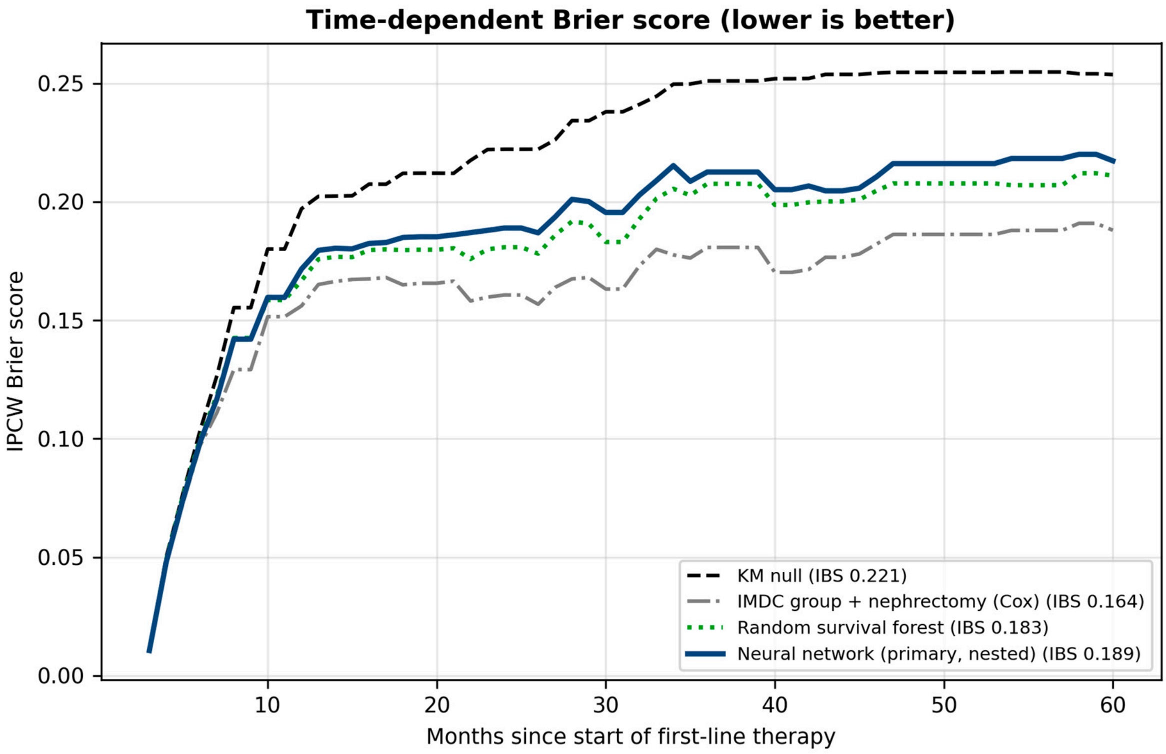

Figure S8. Time-dependent inverse probability of censoring weighted (IPCW) Brier scores for the neural network, the Cox model with IMDC risk group and nephrectomy status, the random survival forest, and the Kaplan–Meier null model, based on nested out-of-fold predictions over 3–60 months; lower values indicate more accurate predicted survival probabilities. Integrated Brier scores (IBS) are given in the legend.
